# Mn^2+^ and [Ru(bpy)_3_]^2+^ in UiO-67 metal organic frameworks enhance photocatalytic oxidation of benzylamine *via* an electron transfer pathway

**DOI:** 10.1039/d5ra04503g

**Published:** 2025-10-13

**Authors:** Subrata Mandal, Novitasari Sinambela, Johannes Biskupek, Riccarda Müller, Ute Kaiser, Kerstin Leopold, Andrea Pannwitz

**Affiliations:** a Institute of Inorganic Chemistry I, University Ulm Albert-Einstein-Allee 11 89081 Ulm Germany; b Central Facility of Electron Microscopy, Electron Microscopy Group of Material Science, University of Ulm Albert-Einstein-Allee 11 Ulm 89081 Germany; c Institute of Analytical and Bioanalytical Chemistry, University Ulm 89081 Ulm Germany; d Institute for Inorganic and Analytical Chemistry, Friedrich-Schiller-University Jena Humboldtstr. 8 07743 Jena Germany Andrea.pannwitz@uni-jena.de; e Center for Energy and Environmental Chemistry Jena (CEEC), Friedrich-Schiller-Universität Jena Philosophenweg 7a 07743 Jena Germany; f Helmholtz Institute for Polymers in Energy Applications Jena (HIPOLE Jena) Lessingstraße 12-14 07743 Jena Germany

## Abstract

The selective oxidation of amines to imines is an essential transformation in many chemical syntheses. Driving such reactions with light and earth abundant catalysts is highly interesting in the context of solar light energy conversion and broadening the reaction scope in organic synthesis. The here reported bimetallic metal–organic framework (MOF), UiO-67 Ru_50_-Mn_10_, allows oxidation of benzylamine under ambient conditions *via* dual electron and energy transfer with oxygen. UiO-67 Ru_50_-Mn_10_ integrates [Mn(bpy)_2_(Cl)_2_] active sites and light-active [Ru(bpy)_3_]^2+^ photosensitizers at the linkers of the UiO-67 scaffold. The spatial organisation of photo and redox-active centres enables efficient charge separation and mass transport. Under 460 nm LED irradiation for 24 h and ambient aerobic conditions, UiO-67 Ru_50_-Mn_10_ catalyses the oxidation of benzylamine to *N*-benzylidene-1-phenylmethanamine with turnover numbers up to 634, which is 1.6 to 1.8 times higher than homogeneous analogues and the Mn-free UiO-67 Ru_50_. Structural and spectroscopic studies confirm successful incorporation of both metals, while mechanistic analyses reveal dual electron and energy transfer pathways, influenced by the solvent environment. This work highlights the potential of heterometallic MOFs with earth-abundant catalytic sites as efficient platforms for photocatalytic oxidative transformations.

## Abstract 2

A UiO-67 MOF functionalized with [Ru(bpy)_3_]^2+^ and Mn^2+^ ions is active in light-driven amine oxidation. The oxidation *via* photosensitized singlet oxygen and electron transfer to manganese was evaluated.

## Graphical abstract

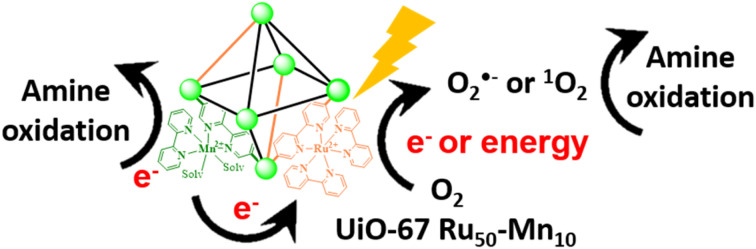

## Introduction

The selective oxidation of amines to imines is an essential transformation in organic synthesis, with applications in fine chemicals, pharmaceuticals, and industrial catalysis. Traditionally, these reactions require harsh oxidants and elevated temperatures, making the development of efficient, sustainable, and photocatalytic alternatives highly desirable.^1^ Among various traditional photocatalytic systems, such as TiO_2_ and CdS, which have poor structure adjustment and low photocatalytic efficiency,^2,3^ metal–organic frameworks (MOFs) have gained significant attention due to their tunable porosity, structural flexibility, and ability to integrate very specific molecular photosensitizers (PSs) and catalytic centres (CATs) within a single platform.^4^ Considering the success of MOFs in artificial photosynthesis,^5,6^ significant efforts have focused on using them as carriers for catalysts, photosensitizers (PSs), or both, to enhance charge transfer and unlock new catalytic pathways for improved efficiency and selectivity in photocatalytic oxidation reactions.^7^ Additionally, the heterogeneous nature of MOF-based systems offers superior recyclability and stability, which are often challenging to achieve with their homogeneous counterparts.^5^

For example, Li and co-workers reported the oxidation reactions of a series of amines to imines with conversion yields of around 41–99% and selectivity of around 45–90% using molecular O_2_ as an oxidant over the MOF NH_2_-MIL-125(Ti) under visible light irradiation.^8^ Bai *et al.* reported that the MOF LTG-NiRu, constructed from a [Ru(phen)_3_]^2+^-derived linker and Ni^2+^-functionalized nodes, exhibited exceptional catalytic activity in the aerobic photocatalytic oxidative coupling of amine derivatives, achieving nearly 100% conversion of benzylamines within 1 h under visible light.^9^ Jin and co-workers developed a mixed-linker Zr-MOF incorporating both electron donor and acceptor linkers based on naphthalene diimide and porphyrin, which showed high photocatalytic activity for aerobic oxidative coupling of benzylamine.^10^ However, in such systems, catalytic activity or charge separation often occurs between the parent linker and the secondary building unit (SBU), which can result in partial framework degradation.^11^ Although the latter one is stable, it lacks defined active sites and selective reaction pathways.

Incorporation of PS and single-site catalysts into a MOF represents a very interesting field for other photocatalytic reactions, such as the H_2_ evolution reaction (HER),^12,13^ CO_2_ reduction reaction (CO_2_RR),^14–17^ and alcohol oxidation,^18^ however, their application related to benzylamine oxidation is rarely explored.

Nature's Photosystem II (PS II) provides a blueprint for light-driven, redox chemistry through the precise arrangement of a chlorophyll-based photosensitizer (P680) and a Mn_4_CaO_5_ cluster that catalyses water oxidation.^19^ While synthetic molecular models of PSII have largely aimed to replicate water oxidation,^20^ often using Ru-based photosensitizers linked to manganese centres,^21,22^ the potential of such systems for alternative oxidative transformations remains underexplored.

In this study, a Ru(bpy)_3_^2+^-functionalized MOF (UiO-67 Ru_*x*_) was synthesized and further modified by coordinating Mn^2+^ sites *via* post-synthetic modification (PSM) to the bipyridine linkers of the framework, yielding the bimetallic system UiO-67 Ru_*x*_-Mn_*y*_. The concept is based on leveraging the structural stability and modularity of the UiO-67 backbone to organize photoactive ruthenium complexes and redox-active manganese centres in proximity, mimicking the compartmentalized environment of natural photosystem II. The resulting hybrid material was explored as a visible-light-responsive catalyst for the oxidative conversion of benzylamine to imine in aerobic conditions, and its activity was benchmarked against related systems, mono-functionalized analogues (UiO-67 Ru and UiO-67 Mn), and homogeneous reference complexes such as Ru(bpy)_3_^2+^ and Mn(bpy)_2_Cl_2_. To validate successful Ru and Mn integration and preserve framework structure, multiple analytical tools were employed, including X-ray diffraction (XRD), infrared (IR) spectroscopy, nuclear magnetic resonance (NMR) spectroscopy, transmission electron microscopy (TEM), and X-ray photoelectron spectroscopy (XPS). To probe charge separation and electron-transfer behaviour, a combination of steady-state and time-resolved spectroscopic techniques, UV-vis absorption, photoluminescence quenching, and time-resolved emission, was employed, along with electrochemical studies.

This work sheds light on the cooperative effects between integrated light-absorbing and catalytic centres in MOF-based systems, offering new directions for the rational design of high-performance photocatalytic platforms for oxidative reactions.

## Results and discussion

### Synthesis and characterization

**Fig. 1:**
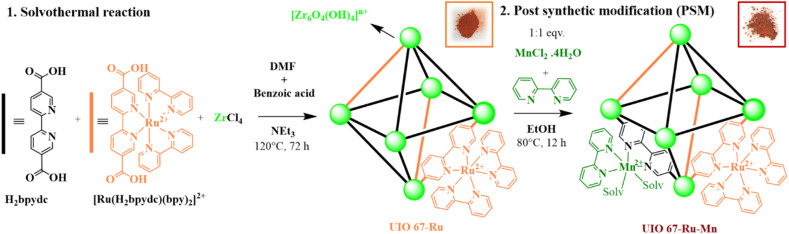
Synthetic procedure of UiO-67 Ru-Mn *via* solvothermal synthesis and PSM.

The UiO-67 MOFs of 2,2′-bipyridine-5,5′-dicarboxylate (bpydc)-based linker functionalized with Ru-based PS and single-site Mn catalyst were achieved *via* a two-step self-assembly process to yield composite photocatalytic systems (UiO-67 Ru_*x*_-Mn_*y*_) with an elemental ratio of Ru/Mn (*x* : *y* = 5 : 1 and 1 : 1). In these processes, the MOFs UiO-67 Ru_*x*_-Mn_*y*_ (10 : 10 and 50 : 10) were designed and synthesized by a mixed-ligand synthetic strategy to introduce the Ru-units, and subsequently post-synthetic metalation (PSM) to introduce the Mn-sites. The metallo-ligand linker [Ru(H_2_bpydc)(bpy)_2_]Cl_2_ was prepared according to the previously published method.^23^ The red coloured MOFs UiO-67 Ru_10_ and UiO-67 Ru_50_ (10 and 50% Ru respective to the bpdyc) were synthesized by carefully adjusting the ratio of 2,2′-bipyridine-5,5′-dicarboxylic acid (H_2_bpydc) and [Ru(H_2_bpydc)(bpy)_2_]Cl_2_ to react with ZrCl_4_ under solvothermal conditions and in presence of benzoic acid (BA) in *N*,*N*-dimethylformamide (DMF) at 120 °C (Fig. 1).^24^ To generate control samples, white colour UiO-67 was synthesized by using only the H_2_bpydc. The PSM of UiO-67 Ru_*x*_ and UiO-67 were performed by treating those nanocrystals with feed amounts of 10% (respective to the bpdyc) MnCl_2_ and 10% additional bipyridine (bpy) solution in ethanol (Fig. 1).

**Fig. 2:**
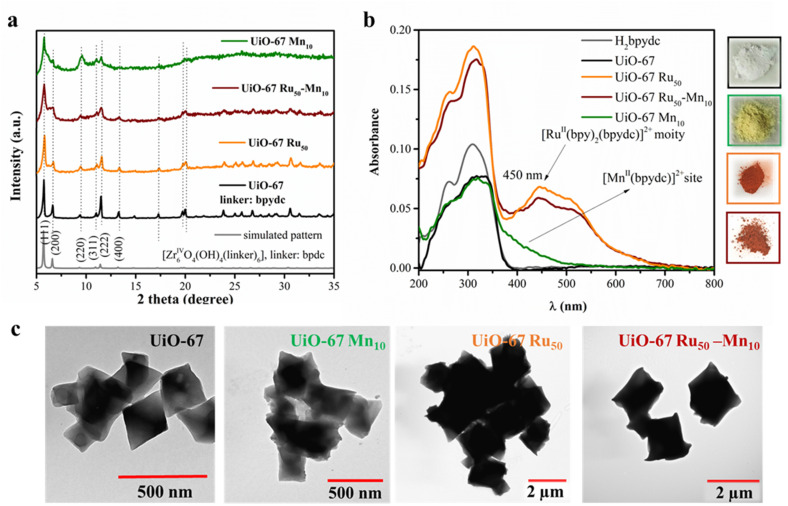
(a) PXRD patterns of UiO-67 Ru_50_-Mn_10_ and their counterparts (UiO-67, UiO-67 Mn_10_, UiO-67 Ru_50_) and the simulated XRD pattern of UiO-67 made up of biphenyl dicarboxylate (bpdc) linker obtained from the crystallographic data reported earlier,^25^ and their (b) UV-vis absorption spectra obtained from the solid-state diffuse reflectance spectroscopy. Inset on the right side of the figures shows the photograph of the prepared samples. (c) Transmission electron microscopic (TEM) images of the UiO-67, UiO-67 Mn_10_, UiO-67 Ru_50_, and UiO-67 Ru_50_-Mn_10_ (from left to right). The colour code of the MOFs is as follows: black = UiO-67, green = UiO-67 Mn_10_, orange = UiO-67 Ru_50_, and brown = UiO-67 Ru_50_-Mn_10_.

The crystallinity of these UiO-67 MOFs was maintained well in UiO-67-Ru_*x*_ and UiO-67 Ru_*x*_-Mn_10_ and UiO-67 Mn_10_, which was confirmed by PXRD patterns similar to that of the here synthesized reference UiO-67 (Fig. 2a). Moreover, each phase exhibited characteristic reflection planes (111), (200), (220), (311), (222), and (400) at angles 5.7°, 6.6°, 9.3°, 10.9°, 11.4°, and 13.2°, respectively, confirming that they are isostructural to the reported face-centred cubic phase of UiO-67 (bpdc) (bpdc = biphenyl dicarboxylate) with the space group *Fm*3̄*m*.^25^ This unique topology of UiO-67 (Fig. S5) has a periodic structure with an ideal unit cell formula of Zr_6_O_4_(OH)_4_(BPDC)_6_, where this cluster adopts an octahedral geometry with six Zr(iv) cations occupying its vertices.^26^ Successful incorporation of Ru and Mn moieties was supported by diffuse reflectance UV/vis spectroscopy. A broad absorption (∼400–700 nm) in the spectrum of UiO-67 Ru_50_-Mn_10_ was consistent with the absorption of UiO-67 Ru_50_, which can be attributed to the metal-to-ligand charge transfer (MLCT) absorption of [Ru(bpy)_3_]^2+^ moiety in the UiO-67 framework (Fig. 2b). An increase in the absorption edge and blue shift of the highest absorption MLCT band compared to spectra of homogenous [Ru(H_2_bydc)(bpy)_2_]^2+^ (Fig. S2) can be attributed to the immobilization effect of the complex in a rigid environment.^27^ The immobilization of the Mn complex, however, is not distinguishable, but can be supported by studying the diffuse reflectance UV-visible spectroscopy of the control UiO-67 Mn_10_ counterparts. As shown in Fig. 2b, an additional broad absorption in the range of 360–500 nm in UiO-67 Mn_10_ is observed compared to that of pristine UiO-67, resulting in a yellow colored solid (Fig. 2b, inset), which can be attributed to the absorption resulting from the Mn-complex.^28^ Compared to the spectra of its homogeneous molecular model [Mn(bpy)_2_Cl_2_] (Fig. S2), a significant red shift in the MOF can be ascribed to the role of the solvent effect on the absorption band, tentatively assigned to an MLCT.^28^

The TEM images of UiO-67 Ru_50_ and UiO-67 Ru_50_-Mn_10_ (Fig. 1c) reveal that both samples exhibit a well-defined octahedral crystalline morphology, with crystal sizes ranging from approximately 1 to 2 μm. Notably, no significant morphological changes are observed when compared to their UiO-67 and UiO-67 Mn_10_ counterparts. However, a pronounced difference in crystal size is evident: UiO-67 and UiO-67 Mn_10_ crystals are considerably smaller, typically ranging from 200 to 500 nm. This variation in crystal size is likely attributed to differences in reaction kinetics between Zr(iv) and the H_2_bpydc linker *vs.* the [Ru(H_2_bpydc)(bpy)_2_]Cl_2_ complex. Additionally, factors such as linker acidity, solubility, and geometry, which are altered upon metalation, can significantly affect the nucleation and growth processes during synthesis, ultimately influencing the final crystal size.^29^ Notably, in both cases, post-synthetic modification (PSM) for Mn incorporation does not result in any observable changes in crystal size or shape, indicating that the overall framework integrity remains unaffected.

**Fig. 3:**
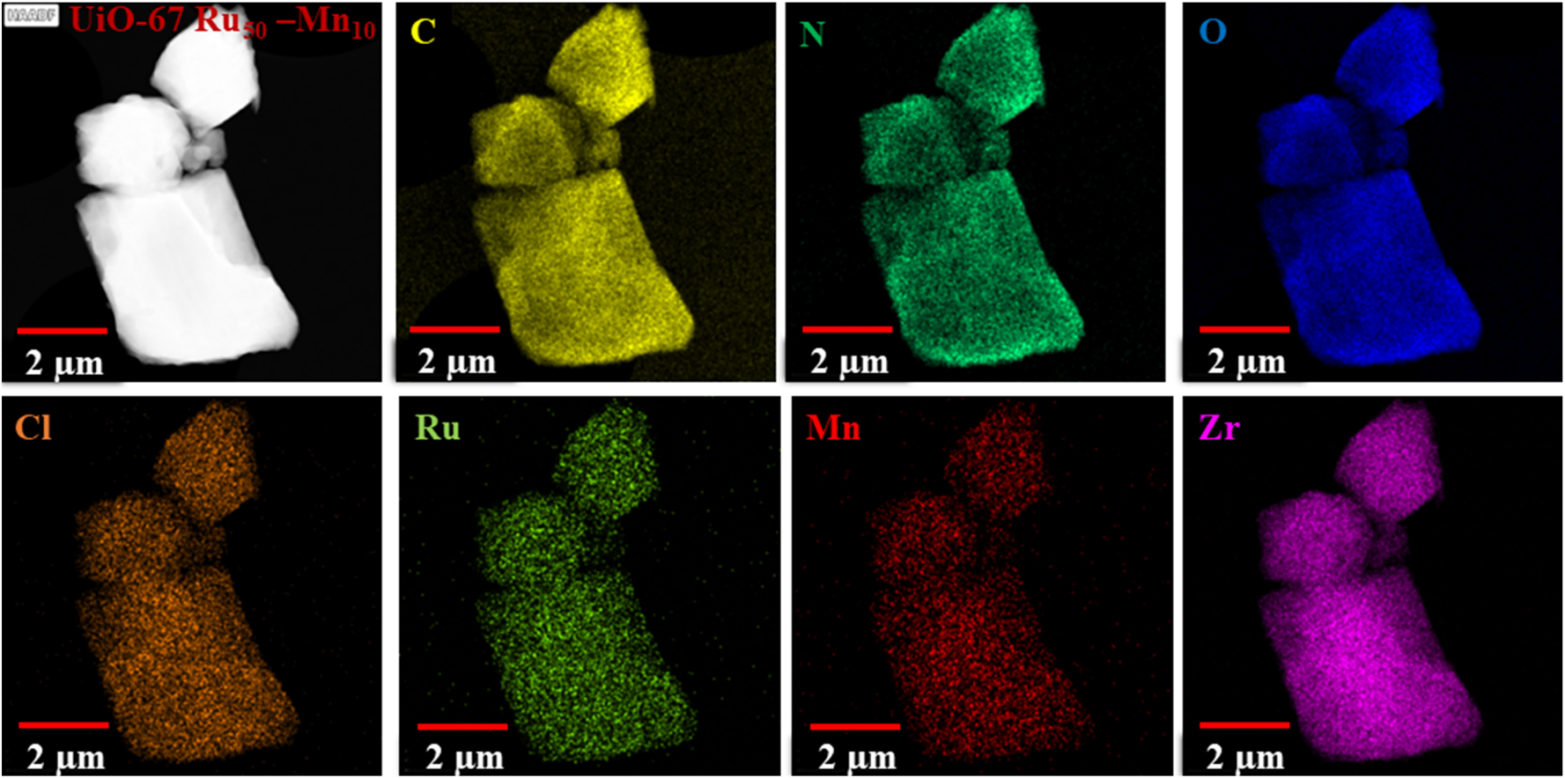
High-angle annular dark field scanning transmission electron microscopic (HAADF-STEM) image with corresponding EDX element mapping images of UiO-67 Ru_50_-Mn_10_. The yellow, dark green, blue, orange, light green, red, and purple signals represent C, N, O, Cl, Ru, Mn, and Zr, respectively. Please note, that the Mn-mapping is mostly an artifact due to overlapping of Mn-Kβ lines with Fe-Kα lines that originate from stray electrons on the TEM column made of iron.

High-angle annular dark-field scanning transmission electron microscopy (HAADF-STEM) images together with corresponding energy dispersive X-ray (EDX) element mappings of UiO-67 Ru_50_-Mn_10_ are shown in Fig. 3 and S6, S7, SI, for UiO-67 Ru_50_, respectively. The HAADF-STEM and EDX images clearly show the presence of elements Zr, C, N, O, Cl, and Ru, which are distributed throughout the entire structure of each MOF. The signals for Mn were detected in UiO-67 Ru_50_-Mn_10_. Unlike C, N, O, and Zr in both the framework, the Ru, Cl in both the framework, and Mn metals in UiO-67 Ru_50_-Mn_10_ appear to be more spatially isolated. This observation may be attributed either to the separation of individual metal-complex centers within the linker motif, as enforced by the framework structure, or to the relatively low composition of these elements in the overall material. Notably, the EDX spectra of these MOFs (Fig. S6, S7 and Table S2, SI) confirm the presence of Ru and Cl signals; however, the Mn signal observed in UiO-67 Ru_50_-Mn_10_ arises from the overlap of the Mn Kβ line with the Fe Kα line, where Fe originates from stray electrons scattered on the TEM column made of iron. Consequently, the Mn elemental map should largely be regarded as an artifact. The experimentally determined average Zr/Ru ratios for UiO-67 Ru_50_ and UiO-67 Ru_50_-Mn_10_ are ∼1.12–6.35 and ∼11.4, respectively. These values deviate significantly from the expected stoichiometric ratio of Zr/Ru = 2, according to the ideal formulas: UiO-67 Ru_50_ ([Zr_6_O_4_(OH)_4_(bpydc)_3_]·3[Ru(bpy)_2_(bpydc)]Cl_2_) and UiO-67 Ru_50_-Mn_10_ ([Zr_6_O_4_(OH)_4_(bpydc)_2.4_]·3[Ru(bpy)_2_(bpydc)]Cl_2_·0.60[Mn(bpy)(bpydc)Cl_2_]). This discrepancy suggests that the distribution of Ru within the framework is random and that both MOFs deviate from their idealized formulas, either due to residual solvent molecules from the synthesis or the presence of linker vacancies.

The chemical compositions of UiO-67 and UiO-67-Ru_*x*_ were determined by ^1^H-NMR measurements in DMSO-d_6_ by digesting them in DCl/D_2_O (see SI page 8–9 for detailed calculations). A previous report had suggested that benzoic acid is commonly entrapped within the pore of the MOF and, consequently, cannot be completely removed during the washing process.^30^ In our case, the ^1^H-NMR spectrum displayed similar signals corresponding to benzoate, while the presence of a negligible amount of formate (HCOO^−^) is there, although formate was not used in the initial synthesis, this molecule was produced due to the decomposition of DMF during the solvothermal reaction (Fig. S8).^30^ These molecules likely participate in the Zr-cluster assemblies similar to some Zr-MOFs, where the modulators were identified in the crystal structures.^30,31^ As the formate peak is negligible it is ignored for the current analysis. Based on the integration of the signals of BA, including the bpydc, the chemical formula of UiO-67 was estimated to be [Zr_6_O_4_(OH)_4_(bpydc)_5.82_(BA)_0.36_] (Table S3). In UiO-67 Ru_50_ and UiO-67 Ru_10_, the [Ru(bpy)_2_(bpydc)]Cl_2_ moiety can be characterized by ^1^H NMR, and analysis was possible because it remains intact under these MOF digestion conditions (Fig. S8). Based on the integration of the proton resonances for [Ru(bpy)_2_(bpydc)]Cl_2_ in UiO-67 Ru_50_, and UiO-67 Ru_10_ the chemical formula was estimated to be [Zr_6_O_4_(OH)_4_(bpydc)_4.724_(BA)_1.13_]·0.7[Ru(bpy)_2_(bpydc)]Cl_2_, and [Zr_6_O_4_(OH)_4_(bpydc)_5.48_(BA)_0.71_]·0.16[Ru(bpy)_2_(bpydc)]Cl_2_, respectively, and the degree of Ru modification in UiO-67 Ru_*x*_ MOFs is 13% and 3%, respectively, indicating that complete incorporation of the Ru moiety did not take place. Unfortunately, the quantification of Mn quantification *via* NMR is not possible, as Mn^2+^ is paramagnetic, resulting in a broad signal as observed in UiO-67 Ru_10_-Mn_10_ (Fig. S8).

**Fig. 4:**
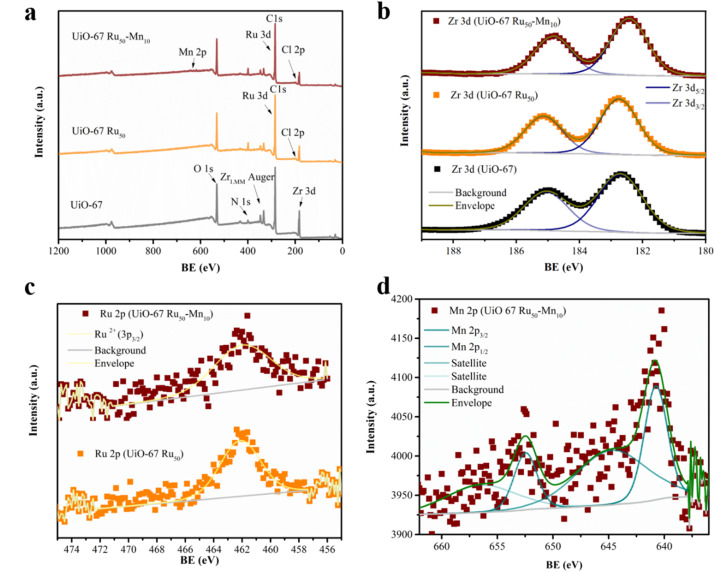
X-ray photoelectron spectra (XPS) of UiO-67-based materials: (a) survey spectra of UiO-67, UiO-67 Ru_50_, and UiO-67 Ru_50_-Mn_10_; (b) high-resolution Zr 3d spectra for all three samples; and (c) high-resolution Ru 2p spectra for UiO-67 Ru_50_ and UiO-67 Ru_50_-Mn_10_ and (d) Mn 2p for UiO-67 Ru_50_-Mn_10_. BE = binding energy.

X-ray photoelectron spectroscopy (XPS) was used to determine the precise elemental composition, the integrity of the Ru and Mn complexes, and their electronic states within the MOFs (Fig. 4a). All shifts for the samples were corrected by normalizing the C (1s) binding energy to 284.8 eV (Fig. S9a). In all the MOFs, including C 1s, all characteristic peaks for Zr 3d at 180.95–187.05 eV (Fig. 4b), N 1s at 395.90–402.30 eV (Fig. S9b), and O 1s at 528.00–535.50 eV^32,33^ (Fig. S9c) were observed, corresponding to the primary backbone (linker and SBU), and notably, slight changes are observed in UiO-67-Ru_50_, and UiO-67 Ru_50_-Mn_10_ MOFs for the local environment exerted by the Ru and Mn molecular moieties. For instance, the binding energy of N 1s in UiO-67 is 398.2 eV, which is attributed to N in bpydc^2−^ (Fig. S9b). The electron density of nitrogen at this region was reduced, followed by the formation of coordination interactions between bpydc^2−^ and the Ru^2+^ and Mn^2+^ species in UiO-67 Ru_50_ and UiO-67 Ru_50_-Mn_10_, leading to distinct N 1s peaks at 398.6 eV and 399.00 eV, respectively. The change in the peak of N 1s to higher binding energy indicates that the N atom on the bipyridine (bpydc^2−^) is coordinated with the Ru ion in UiO-67 Ru_50_ and Ru and Mn ions, which reduces the electron density at the N atom.^34,35^ Additionally, in contrast to UiO-67, for both UIO-67 Ru_50_ and UIO-67 Ru_50_-Mn_10_, two peaks are apparent in the Ru regions at 285.37 and 281.12 eV, which are assigned to Ru^2+^ 3d_3/2_ and 3d_5/2_, respectively (Fig. S9a) which almost overlaps with the C 1s signal (Fig. 4a). Fig. 4c shows the respective peaks at around 462.12 eV assigned to the Ru 2p_3/2_ region and, which are in good agreement with the pattern of divalent ruthenium, Ru^2+^.^12^ No other valence state peaks, such as Ru^3+^ or Ru^4+^, were detected. The Mn 2p spectra (Fig. 4d) observed in UiO-67 Ru_50_-Mn_10_ contain two main peaks centred at 640.75 eV (Mn 2p_3/2_) and 652.5 eV (Mn 2p_1/2_), while no Mn^3+^ and Mn^4+^ species are observed, which are generally observed at higher energies than the Mn^2+^ species. Additionally, the spectra consist of satellite peaks with considerable intensity at ∼5 eV higher binding energy (645.10 eV) compared to that of the corresponding main Mn 2p_3/2_ peak. These spectral features reflect that the valence of the central manganese ion in the UiO-67 Ru_50_-Mn_10_ is +2 (ref. 36 and 37) and [Mn(bpy)(bpydc)Cl_2_] remained stable during the PSM process. It should be noted that the Cl 2p peaks in UiO-67 Ru_50_ and UiO-67 Ru_50_-Mn_10_ MOFs at around 195.25–201.62 eV exist (Fig. S9d), representing Cl anion as a counter anion.

Despite the presence of the Ru peak being detected in UiO-67 Ru_50_-Mn_10_, Ru was not taken into account for estimating the Ru/Mn ratio due to the overlap with C and a weak signal. Therefore, the Zr/Mn ratio was considered instead, which was found to be 7.73 in UiO-67-Ru_50_-Mn_10_. Combined with the Zr : Ru ratio of 8.57 obtained from NMR analysis of UiO-67-Ru_50_ (assuming no leaching occurred during the post-synthetic modification, PSM), these measurements allowed the estimated atomic ratio of Zr : Ru : Mn to be calculated as 1 : 0.117 : 0.129. Based on this, the proposed chemical formula of UiO-67 Ru_50_-Mn_10_ is: [Zr_6_O_4_(OH)_4_(bpydc)_3.944_(BA)_1.13_]·0.70[Ru(bpy)_2_(bpydc)]Cl_2_·0.78[Mn(bpy)(bpydc)Cl_2_]. In accordance with the above formulation, the molar Ru/Mn ratio is nearly 0.89. This ratio is expected to be reflected in the high resolution-continuum graphite furnace atomic absorption spectrometry (HR-CS-GFAAS) and total X-ray fluorescence spectrometry (TXRF) measurements of the MOF (see SI for details and Fig. S10). Indeed, the element analysis in Table S5 confirms that the Ru/Mn molar ratio is ∼1.02, providing excellent corroboration of the structural formulation.

### Photocatalytic activity for the oxidation of benzylamine

The photocatalytic oxidation of benzylamine was carried out under ambient conditions using the synthesized MOFs as catalysts. Reactions were performed in a mixed solvent system of acetonitrile : H_2_O (4 : 1 v/v) under an air atmosphere. The reaction setup utilized a 3D-printed modular photoreactor equipped with a 460 nm LED light source (800–1250 mW) operating at a current of 0.7 A and a voltage of 4 V.^27,30^ The reaction progress was initially monitored under radiation for 36 h (Fig. S11), and the imine product *N*-benzylidene-1-phenylmethanamine formed after 24 h for each set of reactions were quantified using ^1^H NMR spectroscopy, with 1,3,5-trioxane employed as an internal standard (see SI page 17 for details and Fig. S12), and the yields (%) calculated for the conversion to *N*-benzylidene-1-phenylmethanamine are listed in Table 2.

**Table 1:** Calculated formulas of the MOFs, according to ^1^H NMR^a^

Samples	Calculated formula of the MOF [Zr_6_O_4_(OH)_4_(bpydc)_*x*_(BA)_*y*_]
UiO-67	[Zr_6_O_4_(OH)_4_(bpydc)_5.82_(BA)_0.36_]
UiO-67 Ru_50_	[Zr_6_O_4_(OH)_4_(bpydc)_4.724_(BA)_1.13_]·0.70[Ru(bpy)_2_(bpydc)]Cl_2_
UiO-67 Ru_10_	[Zr_6_O_4_(OH)_4_(bpydc)_5.48_(BA)_0.71_]·0.16[Ru(bpy)_2_(bpydc)]Cl_2_
UiO-67 Ru_50_-Mn_10_	[Zr_6_O_4_(OH)_4_(bpydc)_3.944_(BA)_1.13_]·0.70[Ru(bpy)_2_(bpydc)]Cl_2_·0.78[Mn(bpy)(bpydc)Cl_2_]

a The ratio of Ru and Mn in UiO-67 Ru_50_-Mn_10_ MOF was calculated from the combination of ^1^H NMR and XPS data in Tables S3 and S4.

**Table 2:** Photocatalytic benzylamine oxidative coupling reaction^a^

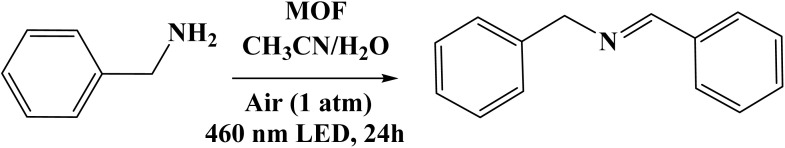							
#	Catalysts	Conditions	Yield^b^ (%)	Selectivity^b^ (%)	TON^c^	TOF^c^ (h^−1^)	Rate (μmol g_MOF_^−1^ h^−1^)
1	UiO-67	CH_3_CN/H_2_O 6 h	1.0	81	—	—	157
2	UiO-67 Mn_10_	CH_3_CN/H_2_O 6 h	0.2	76	—	—	28
3	UiO-67 Ru_50_	CH_3_CN/H_2_O 24 h	27.6	75	358	14.9	4218
4	UiO-67 Ru_50_-Mn_10_	CH_3_CN/H_2_O 24 h	45.1	85	634	26.4	6895
5	UiO-67 Ru_50_-Mn_10_	CH_3_CN/H_2_O 24 h, dark	—	—	—	—	—
6	No catalyst	CH_3_CN/H_2_O 6 h	—	—	—	—	—
7	UiO-67	CH_3_CN, 24 h	1.4	77	—	—	213
8	UiO-67 Mn_10_	CH_3_CN, 24 h	0.7	71	—	—	101
9	UiO-67 Ru_50_	CH_3_CN, 24 h	11.9	93	154	6.4	1822
10	UiO-67 Ru_50_-Mn_10_	CH_3_CN, 24 h	14.6	94	205	8.5	2229
11	UiO-67 Ru_50_-Mn_10_	CH_3_CN/H_2_O 24 h, Ar	—	—	—	—	—
12	UiO-67 Ru_10_	CH_3_CN, 24 h	4.7	80	240	10.0	721
13	UiO-67 Ru_10_-Mn_10_	CH_3_CN, 24 h	3.7	79	193	8.1	564
14	[Ru]	CH_3_CN/H_2_O 24 h	15.7	88	206	8.6	—
15	[Ru] + [Mn]	CH_3_CN/H_2_O 24 h	29.1	93	382	15.9	—
16	[Ru]	CH_3_CN, 24 h	49.5	94	648	27.8	—
17	[Ru] + [Mn]	CH_3_CN, 24 h	58.2	99	763	31.0	—

a Reaction condition: MOF (1 mg), CH_3_CN : H_2_O (4 mL, 4 : 1 v/v) or pure CH_3_CN, benzylamine (80 μL, 0.734 mmol), (*λ* = 460 nm LED, 24 or 6 h), air (1 atm).

b Determined by ^1^H NMR using 1,3,5-trioxane as the internal standard.

c For TON and TOF calculation Ru content was calculated from their respective MOF (1 mg) as per structure formula listed in Table 1. For homogeneous conditions: [Ru] = [Ru(bpy)_2_(bpydc)]Cl_2_, [Mn] = [Mn(bpy)_2_Cl_2_]·H_2_O, 0.28 μmole of [Ru] was used.

**Fig. 5:**
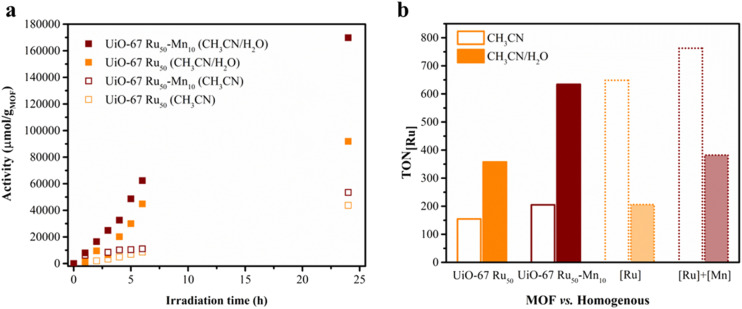
(a) Photocatalytic activity (expressed as μmol g_MOF_^−1^*vs.* time) for the oxidation of benzylamine in the presence of UiO-67 Ru_50_ (orange) and UiO-67 Ru_50_-Mn_10_ (red) conducted in CH_3_CN (hollow square) and CH_3_CN/H_2_O (v/v 4 : 1) (solid square). (b) A comparison of TON_[Ru]_ for the photocatalytic oxidation of benzylamine after 24 h of irradiation for the MOFs with homogeneous [Ru]: [Ru(bpy)_2_(bpydc)]Cl_2_ alone (transparent orange) and [Ru] + [Mn] ([Ru(bpy)_2_(bpydc)]Cl_2_ with [Mn(bpy)_2_Cl_2_]·H_2_O) (transparent red), conducted in CH_3_CN (hollow square) and CH_3_CN/H_2_O (v/v 4 : 1) (solid square).

The control experiments under dark conditions, in absence of any catalyst or MOF are listed in Table 2 entries 5 and 6 and yielded no detectable oxidation products, confirming the necessity of both light and the MOF catalyst for photocatalytic activity. Among the tested materials, UiO-67-Ru_50_ and UiO-67 Ru_50_-Mn_10_ exhibited significant catalytic activity for the oxidation of benzylamine to the corresponding imine (*N*-benzylidene-1-phenylmethanamine) with yields of 27.6% (selectivity 75%) and 45.1% (selectivity 85%), respectively. Notably, no significant imine products under the light-irradiation were observed for the other two counterparts, UiO-67 (yield = 1%) and UiO-67 Mn_10_ (yield = 0.2%), indicating that both light and the Ru moiety are essential components for catalytic activity. In particular and according to the kinetic study, UiO-67 Ru_50_-Mn_10_ showed superior fast performance with a rate of 6895 μmol g_MOF_^−1^ h^−1^. This rate is enhanced compared to UiO-67 Ru_50_ (4218 μmol g_MOF_^−1^ h^−1^) (Fig. 5a). The enhancement observed upon Mn^2+^ incorporation can be attributed to an improved charge transfer efficiency and the presence of additional catalytic sites facilitated by Mn^2+^ within the framework.

The influence of the solvent environment was tested *via* additional experiments in pure acetonitrile (CH_3_CN). While the overall yield of imine products in photocatalysis with the MOFs was lower in pure CH_3_CN, the observed trend in photocatalytic performance among the different MOFs matches the trend in the solvent mixture CH_3_CN : H_2_O (4 : 1 v/v). Specifically, UiO-67 Ru_50_-Mn_10_ with a yield of 14.6% (selectivity 94%) and rate of 2229 μmol g_MOF_^−1^ h^−1^ continued to outperform UiO-67 Ru (yield: 11.9%, with selectivity 93% and rate: 1822 μmol g_MOF_^−1^ h^−1^), highlighting the robustness of its superior activity regardless of the solvent conditions (Fig. 5a). Interestingly, compared to the CH_3_CN/H_2_O mixture, the oxidation reaction of benzylamine in CH_3_CN alone proceeded with higher selectivity, irrespective of the MOF photocatalysts. In aqueous media, a photocatalytic system can form hydroxyl radicals, which can oxidise the substrate and product indiscriminately, thereby reducing the selectivity of the reaction.^38^

Furthermore, the effect of the Ru loading was examined. At low Ru concentrations, only minimal product formation was observed, with UiO-67-Ru_10_ yielding 4.7% and UiO-67 Ru_10_-Mn_10_ yielding 3.7%. No consistent trend was observed at this range (Table 2), further underscoring the pivotal role of the Ru moiety as the primary photosensitizing centre. These findings highlight the necessity of an optimized Ru loading to achieve effective photocatalysis, emphasizing that suboptimal concentrations result in diminished activity, likely due to insufficient active sites for efficient light absorption. Atmospheric conditions were also found to be critical for the reaction. No product formation was observed when the reaction was conducted under an argon atmosphere (Table 2 entry 11), confirming the requirement of molecular oxygen as the terminal oxidant in the photocatalytic cycle.

To assess the role of the MOF-assembly, the homogeneous system was tested, using the molecular analogues [Ru(bpy)_2_(bpydc)]Cl_2_ ([Ru]) and [Mn(bpy)_2_Cl_2_]·H_2_O ([Mn]) in respective combinations in CH_3_CN/H_2_O (v/v 4 : 1) and pure CH_3_CN. As shown in Fig. 5b and Table 2, both molecular systems exhibit similar activity trends, with [Ru] alone (TON = 206) < [Ru] + [Mn] (TON = 382). However, their corresponding MOF systems, UiO-67-Ru_50_ and UiO-67-Ru_50_-Mn_10_, display approximately 1.6-fold higher TONs (358 and 634, respectively) under identical conditions, highlighting the enhanced performance imparted by the heterogeneous framework.

Interestingly, under pure CH_3_CN conditions, the homogeneous systems exhibited a strong increase in TON (643 for [Ru] alone and 763 for [Ru] + [Mn]). A kinetic analysis also revealed that the reaction plateau is reached more quickly in CH_3_CN than in the CH_3_CN/H_2_O mixture (Fig. S13). This trend is opposite to that observed for their MOF counterparts (Table 2 and Fig. 5b) and might be attributed to differences in reaction pathways influenced by the solvent and the 3D-assembly in the MOF *vs.* the diffusion-controlled process in homogeneous conditions.^39,40^

It is interesting to note that, similar to the MOF experiments, the combination of [Ru] and [Mn] in homogeneous solution outperforms [Ru] alone under all tested conditions, suggesting a synergistic interaction between the Ru and Mn components that is preserved even outside of the MOF matrix. More details of the proposed reaction pathways, including electron transfer between the Ru-photosensitizer and the Mn-unit, are discussed *vide infra*.

### Reaction intermediates

**Fig. 6:**
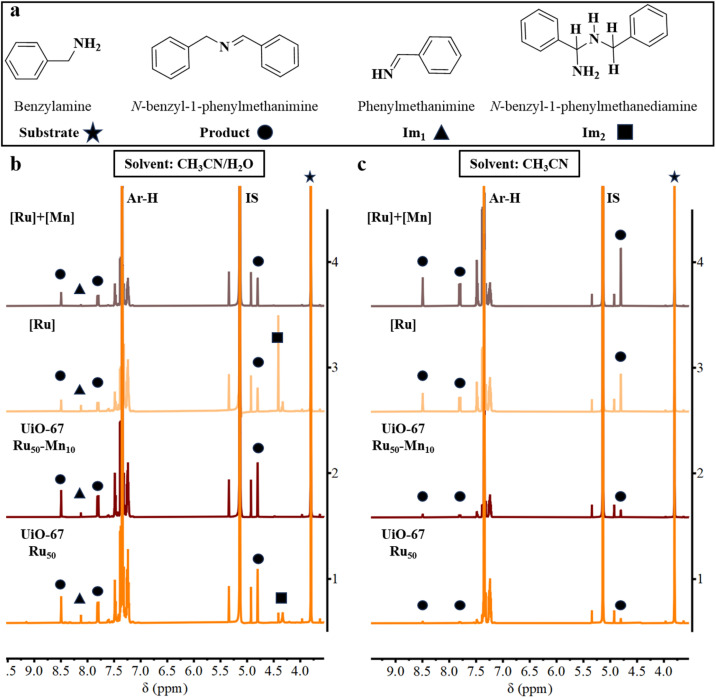
(a) Molecules identified from the ^1^H NMR spectra and (b and c) the corresponding spectra of reaction aliquots (in CD_3_CN) collected at 4 h during the benzylamine oxidation in the presence of UiO-67 Ru_50_, UiO-67 Ru_50_-Mn_10_ MOFs, homogeneous [Ru] ([Ru(bpy)_2_(bpydc)]Cl_2_), and the combination [Ru] + [Mn] ([Ru(bpy)_2_(bpydc)]Cl_2_ with [Mn(bpy)_2_Cl_2_]·H_2_O) under (b) CH_3_CN/H_2_O (v/v 4 : 1) and (c) CH_3_CN solvent conditions. IS = internal standard.

Interestingly, a prominent reaction intermediate phenylmethanimine (Im_1_), along with a tentatively assigned hemiaminal species *N*-benzyl-1-phenylmethanediamine (Im_2_), were detected in the CH_3_CN/H_2_O (v/v 4 : 1) solvent mixture for both the homogeneous [Ru] system and the UiO-67 Ru_50_ catalyst (Fig. 6a and b). In contrast, for UiO-67 Ru_50_-Mn_10_ and the homogenous [Ru] + [Mn] systems, although Im_1_ was identified, the transient intermediate Im_2_ was either absent (UiO-67 Ru_50_-Mn_10_) or detected inconsistently ([Ru] + [Mn]), as indicated by the ^1^H NMR spectra (Fig. 6b and S14–S17). This observation suggests that the hemiaminal species undergoes more rapid decay in the presence of Mn^2+^-incorporated catalysts, particularly UiO-67 Ru_50_-Mn_10_, than in UiO-67 Ru_50_ or the homogeneous [Ru] system. The incorporation of Mn^2+^ likely accelerates the overall reaction rate by promoting a faster conversion of *N*-benzyl-1-phenylmethanediamine to the final imine product, *N*-benzylidene-1-phenylmethanamine. This may occur *via* enhanced electron or proton transfer steps, facilitating more efficient ammonia elimination.

In pure CH_3_CN, no such intermediates were observed for either the MOF-based or homogeneous systems (Fig. 6c and S18–S21), suggesting that an alternative reaction mechanism predominates under non-aqueous conditions. Specifically, energy transfer from the ^3^MLCT (triplet metal-to-ligand charge transfer) state to oxygen, forming the reactive oxygen species ^1^O_2_ can yield to an alternative reaction pathway, followed by rapid condensation of Im_1_ and dehydration of Im_2_, which is likely operative as discussed *vide infra*.

These findings highlight the critical absence or presence of water in modulating the reaction mechanism, modulating both the efficiency and the turnover frequency of the MOF-based catalysts. To further substantiate our observations, we compared the photocatalytic performance of UiO-67 Ru_50_-Mn_10_ with other MOF-based photocatalysts reported previously (Table 3). Since experimental parameters such as initial substrate concentration, catalyst loading, reaction time, and solvent systems vary significantly across studies, we adopted the rate of oxidative coupling (μmol g_MOF_^−1^ h^−1^) in CH_3_CN as a basis for comparison. Compared to benzene dicarboxylate-based and amine functionalized MOFs: UiO-66, UiO-66 NH_2_, and m-NH_2_-MIL-125, n-NH_2_-MIL-125 (Table 3 entries 1–3, and 6), our UiO-67 Ru_50_-Mn_10_ MOF exhibits a rate of 2229 μmol g_MOF_^−1^ h^−1^, which is nearly 16-, 8-, 5-, and 2-fold higher, respectively, under similar solvent conditions. Notably, the rate is quite relatable to the Ru(bpy)_3_@MIL-125 system (Table 3 entry 5). Furthermore, many of the reference MOFs, such as Ti-PMOF-DMA, Zr-NDI-PCOOH, and UiO-68-BT (Table 3 entries 4, 8, and 9), employ novel linker systems and often involve higher synthetic complexity, which may also contribute to their enhanced activity. It is important to note, however, that other experimental factors, particularly the light source characteristics (power and wavelength), were not normalised across these studies, and such variations may exert a significant influence on catalytic performance. Taking this into account, the activity of UiO-67 Ru_50_-Mn_10_ can be considered moderate when compared to those MOFs.

**Table 3:** Reported MOF-based photocatalysts for the oxidative coupling of benzylamine to *N*-benzylidene-1-phenylmethanamine

#	Catalysts	Solvent	Catalyst/benzylamine	Light source, time	Conv. (%)	Selectivity (%)	Yield (%)	Rate (μmol g_MOF_^−1^ h^−1^)
1	UiO-66 (ref. 41)	CH_3_CN	15 mg/0.1 mmol	300 W Xe lamp (no filter), 10 h	42.0	>99	42	140^a^
2	n-NH_2_-MIL-125 (ref. 42)	CH_3_CN	10 mg/0.2 mmol	300 W Xe lamp (no filter), 9 h	98.5	99	97.5	1063
3	m-NH_2_-MIL-125 (ref. 42)	CH_3_CN	10 mg/0.2 mmol	300 W Xe lamp (no filter), 12 h	62.8	92.6	58.15	475
4	Ti-PMOF-DMA^43^	CH_3_CN	5 mg/0.3 mmol	623 ± 8 nm, 0.5 h	94.0	88	82.7	49 620^a^
5	Ru(bpy)_3_@MIL-125 (ref. 44)	CH_3_CN	5 mg/0.1 mmol	Visible light (>440 nm), 3 h	75	>99	74.2	2473^a^
6	UiO-66-NH_2_ (ref. 45)	CH_3_CN	15 mg/0.1 mmol	300 W Xe lamp (no filter), 10 h	83.0	>99	82.2	274
7	LTG-NiRu^46^	DMF	1 mmol%/0.5 mmol	300 W Xe lamp (420–780 nm), 1 h	>99	>99	99%	—
8	Zr-NDI-PCOOH^47^	CH_3_CN	10 mg/0.4 mmol	300 W Xe lamp (full wavelength), 0.75 h	90	100	90	24 000^a^
9	UiO-68-BT^48^	CH_3_CN	5 mg/0.3 mmol	415 nm (3 W × 4), 0.42 h	96	99	95	67 857^a^
10	This work (UiO-67 Ru_50_-Mn_10_)	CH_3_CN/H_2_O	1 mg/0.7 mmol	460 nm (LED 800–1250 mW), 24 h	53	85	45.1	6895
		CH_3_CN			15.5	94	14.6	2229

a The oxidative coupling rate (μmol g_MOF_^−1^ h^−1^) for MOFs is calculated based on their respective catalyst/benzylamine ratio, reaction time and *N*-benzylidene-1-phenylmethanamine yield reported in the literature.

### Recyclability test

The stability of UiO-67 Ru_50_-Mn_10_ during the oxidative coupling of benzylamine was found to be limited to approximately 24 h in a CH_3_CN/H_2_O mixture (Fig. S22). After this period, the solid MOF was almost completely dissolved, as indicated by the appearance of a transparent yellow solution (Fig. S22), which can be attributed to the leaching of photocatalytic components into the solvent. Consequently, the photocatalytic performance of the residual MOF solid recovered after the first cycle was severely diminished. When tested with fresh benzylamine in CH_3_CN/H_2_O (4 : 1), the recovered catalyst yielded only negligible amounts of *N*-benzylidene-1-phenylmethanamine in the second cycle. Such structural collapse of MOFs in aqueous environments is a commonly encountered issue, which may occur either through chemical decomposition or under light irradiation. The dissolution can arise from competitive coordination of ˙OH radicals or amine substrates with the Zr-SBUs, leading to framework breakdown.^38,49^

**Fig. 7:**
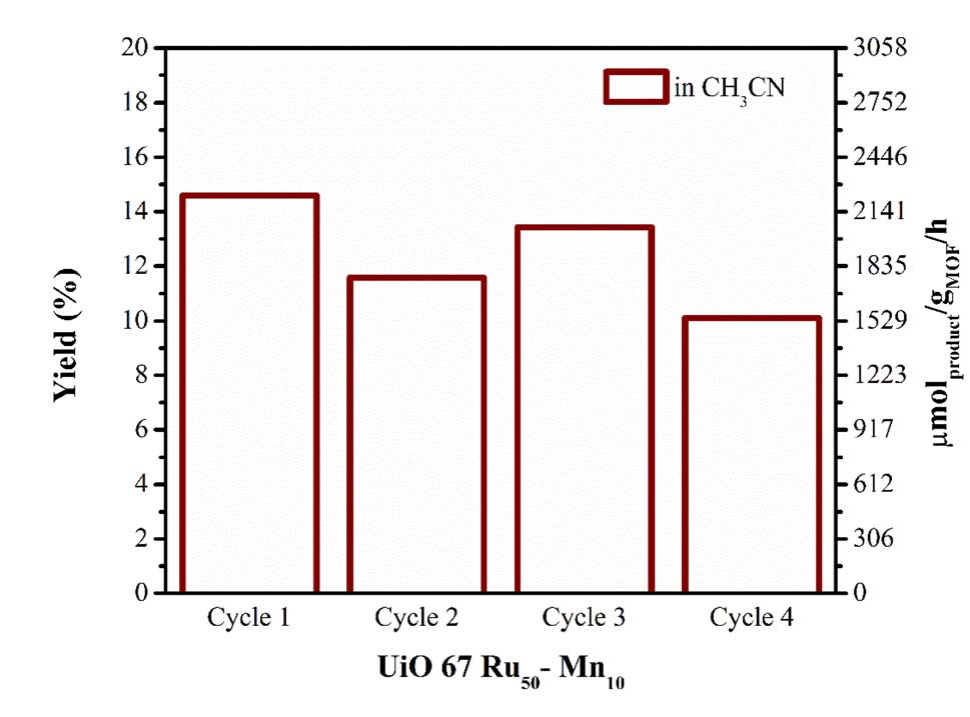
Recyclability test of the UiO-67 Ru_50_-Mn_10_ MOF for the photocatalytic benzylamine oxidative coupling reaction: yield% and μmol g_MOF_^−1^ h^−1^*vs.* time in CH_3_CN (reaction conditions: 1 mg of MOF, *λ*: 460 nm LED source). After each cycle (24 hours of irradiation), the solid was separated from the solution by centrifugation at 7000 rpm, washed with CH_3_CN (twice), and re-irradiated with fresh 0.734 mmol of benzylamine in 4 mL of CH_3_CN.

In contrast, UiO-67 Ru_50_-Mn_10_ exhibited recyclability in pure CH_3_CN (Fig. S23). In this case, the solid MOF could be separated from the reaction medium by centrifugation, washed with CH_3_CN (twice), and reused for further photocatalytic cycles with fresh benzylamine. Although the oxidative coupling of benzylamine in pure CH_3_CN proceeded with lower efficiency (14.6% yield, 2229 μmol g_MOF_^−1^ h^−1^), corresponding to nearly three times lower activity compared to the CH_3_CN/H_2_O system, the catalyst maintained stable performance over multiple cycles without significant loss of photocatalytic activity (Fig. 7).

### Charge and energy transfer dynamics

**Fig. 8:**
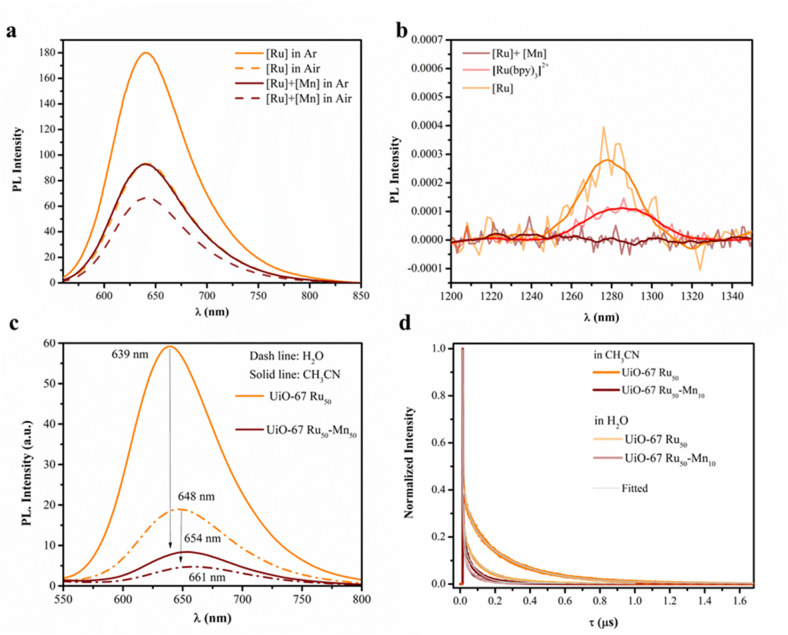
(a) PL spectra of 500 μM [Ru] complex ([Ru(bpy)_2_(bpydc)]Cl_2_) recorded in the 550–800 nm region in the presence and absence of 500 μM [Mn] counterpart ([Mn(bpy)_2_Cl_2_]H_2_O) under both air and Ar atmospheres in CD_3_CN/CD_3_OD (4 : 1 v/v) acquired at *λ*_ex_ = 450 nm. (b) PL spectra of 500 μM [Ru] alone and with 500 μM [Mn], monitored in the 1200–1350 nm region under air in CD_3_CN/CD_3_OD, acquired at *λ*_ex_ = 450 nm. For reference, a standard 500 μM [Ru(bpy)_3_]^2+^ solution in CD_3_CN is included (red solid line). All spectra in (b) were smoothed. (c) PL (*λ*_ex_ = 450 nm) and (d) time-resolved PL (*λ*_ex_ = 372 nm with a 495 nm long pass filter) spectra of UiO-67 Ru_50_ and UiO-67 Ru_50_-Mn_10_ in deaerated CH_3_CN and H_2_O ([MOF] = 0.125 mg mL^−1^).

Oxygen (O_2_) is known as an acceptor to quench the photoexcited photosensitizer *via* energy and/or electron transfer, generating the reactive oxygen species ^1^O_2_ or O_2_˙^−^ respectively.^39,50,51^ To validate these, the [Ru] complex was investigated in homogeneous CD_3_CN/CD_3_OD (4 : 1 v/v) solution by photoluminescence (PL) spectroscopy in the presence and absence of [Mn] counterparts under both air and Ar atmospheres. The ^3^MLCT emission of the photosensitizer at 640 nm and the characteristic ^1^O_2_ emission at 1277 nm were monitored (Fig. 8a and b). The assignment of the ^1^O_2_ emission was confirmed by comparison with the PL spectra of a standard [Ru(bpy)_3_]^2+^ solution in CD_3_CN (Fig. 8b). Consistent with the behaviour of [Ru(bpy)_3_]^2+^ (Fig. S24), the ^3^MLCT emission at 640 nm was significantly quenched under air relative to Ar, by ∼50% for [Ru] alone and ∼30% for the [Ru] + [Mn] system (Fig. 8a). Interestingly, ^1^O_2_ emission at 1277 nm was detected for [Ru] alone, suggesting energy transfer (EnT) from the ^3^MLCT state to ground-state ^3^O_2_. Nevertheless, an electron transfer (ET) pathway from the ^1^MLCT state cannot be excluded, since in the [Ru] + [Mn] system the ^3^MLCT emission was quenched (by ∼30%) without a corresponding increase in ^1^O_2_ generation (Fig. 8b). Additionally, [Mn] itself was found to quench PL of [Ru] by ∼50% even under Ar atmosphere, highlighting its critical role in facilitating ET processes within the [Ru] + [Mn] system under both aerobic and anaerobic conditions.

Therefore, only photoluminescence (PL) spectra and time-resolved PL measurements were conducted on the MOFs to evaluate the charge separation efficiency of photo-excited electron–hole pairs (see SI for experimental details), and all measurements concerning the charge transfer between photoexcited Ru-photosensitizer and Mn were performed under an inert Ar atmosphere. As depicted in Fig. 8c, the MOF sample UiO-67-Ru_50_, containing only the [Ru(bpy)_2_(bpydc)]Cl_2_ photosensitizer, exhibited strong phosphorescence emissions centred at 639 nm in CH_3_CN and 648 nm in H_2_O upon photoexcitation at 450 nm. In contrast, the phosphorescence intensity of UiO-67-Ru_50_-Mn_10_, which incorporates both the PS and [Mn(bpy)(bpydc)Cl_2_] redox-active unit, was significantly reduced in both of these solvents, and consistent with the observations made for the homogeneous [Ru] + [Mn] models.

Time-resolved PL measurements are shown in Fig. 8d and summarized in Table 4, with detailed fitting parameters provided in Table S7. In CH_3_CN, the ^3^MLCT lifetime of the Ru-based photosensitizer decreased from 246 ns in UiO-67 Ru_50_ to 62 ns in UiO-67 Ru_50_-Mn_10_. Similarly, in H_2_O, the lifetime was reduced from 112 ns to 47 ns, as summarized in Table 4. Both of these results demonstrate that the quenching of PS emission in UiO-67 Ru_50_-Mn_10_ arises from accelerated decay of the ^3^MLCT excited state, driven by efficient, solvent-independent interaction and likely reductive electron transfer between the [Ru^II^]* and [Mn^II^] complexes within the MOF framework. However, various studies suggest that, in the presence of external electron acceptors like methyl viologen or O_2_, an oxidative quenching pathway from [Ru^II^]* to [Ru^III^] is followed by electron transfer (ET) from the donor Mn^2+^ in homogeneous systems.^22,52–54^

**Table 4:** Table of photophysical characterization of the [Ru(bpy)_2_(bpydc)]Cl_2_ in homogeneous conditions and UiO-67 Ru_50_ and UiO-67 Ru_50_-Mn_10_ MOF. All measurements were carried out in deaerated solvents. For a homogeneous solution, 50 μM of [Ru(bpy)_2_(bpydc)]Cl_2_ was used

Compounds		^1^MLCT absorption	Optical gap	^3^MLCT emission	
		*λ*_max_ (nm)	*E*_g_ = *E*_HOMO–LUMO_ (eV)	*λ* (nm)	*τ*_av_ (ns)
[Ru(bpy)_2_(bpydc)]Cl_2_	CH_3_CN	460	2.45	615	750
	H_2_O	450	—	670	23
UiO-67 Ru_50_	CH_3_CN	446^a^	2.10^b^	639	246
	H_2_O			648	112
UiO-67 Ru_50_-Mn_10_	CH_3_CN	444^a^	2.10^b^	654	62
	H_2_O			661	47

a Absorption spectra acquired from the diffuse reflectance spectroscopy of the MOF in the solid state.

b Optical energy gaps (*E*_g_) of the solids were estimated from the Kubelka Munk functions.

To evaluate the influence of solvent environment and framework effects, steady-state and time-resolved PL spectroscopy of the [Ru] complex were performed in CH_3_CN and H_2_O under Ar atmosphere and compared with the corresponding measurements for the MOFs. The homogeneous [Ru] complex in CH_3_CN exhibits phosphorescence peaks centred at 615 nm and 670 nm in CH_3_CN and H_2_O, respectively, with corresponding excited-state (^3^MLCT) lifetimes of 750 ns and 23 ns (Fig. S27 and Table 4). Interestingly, both MOF systems in CH_3_CN exhibited significantly shorter ^3^MLCT lifetimes relative to the homogeneous Ru-complex, implying the impact of the MOF platform. However, the homogeneous complex showed a much faster decay in H_2_O, indicating its lower stability of the ^3^MLCT state in aqueous media compared to the MOF systems. These findings highlight two key points: (i) solvent molecules significantly influence the ^3^MLCT state lifetimes, and (ii) the MOF framework provides enhanced stability and lifetime control for the photosensitizer in aqueous environments.

**Fig. 9:**
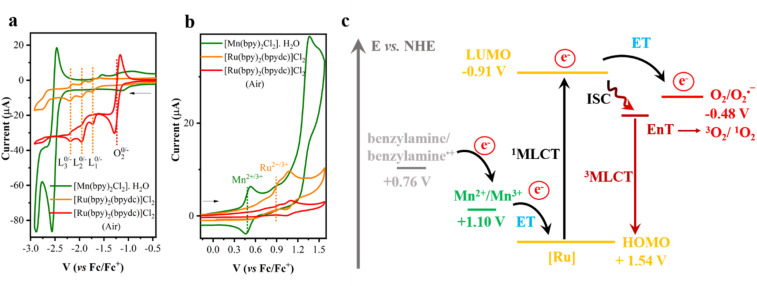
(a and b) Cyclic voltammograms of [Ru(bpy)_2_(bpydc)]Cl_2_ (orange), [Ru(bpy)_2_(bpydc)]Cl_2_ in air (red), and [Mn(bpy)_2_Cl_2_]·H_2_O (green) recorded at a scan rate of 50 mV s^−1^. Experimental conditions: CH_3_CN, 0.1 M *n*Bu_2_NPF_6_ as supporting electrolyte, 0.25 mM analyte concentration, glassy carbon working electrode, Pt wire counter electrode, and Ag/AgCl quasi-reference electrode referenced against Fc/Fc^+^. (c) Schematic energy level diagram illustrating (1) electron transfer (ET) from the excited [Ru(bpy)_2_(bpydc)]Cl_2_ to O_2_, followed by ET from [Mn(bpy)_2_Cl_2_]·H_2_O to the oxidized Ru complex (or *vice versa*), resulting in the formation of superoxide radical (O_2_˙^−^) and (2) singlet oxygen (^1^O_2_) *via* energy transfer (EnT).

Furthermore, cyclic voltammetry (CV) experiments were conducted on the individual complexes in CH_3_CN under both Ar and air atmospheres to gain further insight into the electron transfer processes. In the CV profile of [Ru(bpy)_2_(bpydc)]Cl_2_ (Fig. 9a and S28a), three distinct reduction peaks were observed at −1.60 V, −1.90 V, and −2.13 V *vs.* Fc/Fc^+^, corresponding to the successive reductions of the ligand in Ru-based photosensitizer (PS), as summarized *vs.* NHE in Table S8.^52,55^ An oxidation peak attributed to the Ru^2+/^Ru^3+^ redox couple (HOMO level) was also detected at +0.84 V *vs.* Fc/Fc^+^ (Fig. 9b).^55^ The estimated HOMO and LUMO energy levels are to be +1.53 V, and −0.91 V *vs.* NHE, respectively (Fig. 9c). For [Mn(bpy)_2_Cl_2_]·H_2_O, a well-defined oxidation peak corresponding to the Mn^2+^ to Mn^3+^ transition appeared at +0.41 V *vs.* Fc/Fc^+^ (+1.10 V *vs.* NHE) (Fig. S28b and 9b), which is consistent with previously reported values.^52,54,55^ Notably, this oxidation potential +1.10 V *vs.* NHE is slightly less positive than the estimated HOMO level (+1.53 V *vs.* NHE) of [Ru(bpy)_2_(bpydc)]Cl_2_, indicating a thermodynamically favourable pathway for electron transfer from Mn^2+^ to the oxidized Ru^3+^ species. Under an O_2_ atmosphere, an additional reduction peak attributable to the O_2_/O_2_˙^−^ couple at −1.17 *vs.* Fc/Fc^+^ (−0.48 V *vs.* NHE)^56,57^ was observed in the CV of [Ru(bpy)_2_(bpydc)]Cl_2_ (Fig. 9a and S28c). The LUMO level of the Ru-complex (−0.91 V *vs.* NHE) lies higher in energy than the O_2_/O_2_˙^−^ redox couple (−0.48 V *vs.* NHE), providing a driving force for electron transfer from the excited ^1^MLCT Ru-PS to molecular oxygen (Fig. 9c). These observations suggest that electron transfer from Ru-PS to O_2_, followed by Mn^2+^ to the oxidized Ru centre, or *vice versa*, is both energetically feasible.

**Fig. 10:**
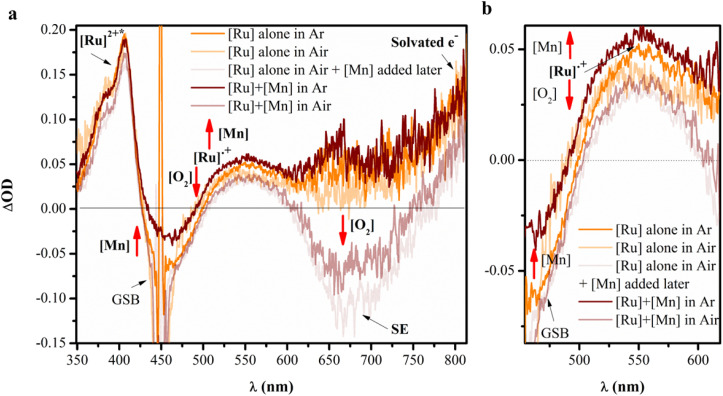
(a) Transient absorption spectra of 500 μM [Ru] complex ([Ru(bpy)_2_(bpydc)]Cl_2_) alone and with 500 μM [Mn] ([Mn(bpy)_2_Cl_2_]·H_2_O) in CH_3_CN/H_2_O (4 : 1 v/v) at a time delay of 100 ns under Ar, and air. (*λ*_pump_ = 450 nm). (b) Enlarged view of the spectra in the 450–610 nm region (GSB: ground state bleach, SE: stimulated emission).

As the ET processes from the excited state are expected to be kinetically fast, we conducted nanosecond transient absorption spectroscopy of the [Ru] complex in CH_3_CN/H_2_O in the presence and absence of [Mn], and under aerobic and anaerobic conditions. The spectra shown in Fig. 10 and S25 were recorded with a delay of 100 ns after the laser pulse (*λ* = 450 nm). For the [Ru] complex alone in aqueous environment under Ar, the spectrum showed a ground-state bleach (GSB) at 450 nm and excited-state absorption at around 400 nm, along with two new positive absorption features centred at around 550 nm and around 800 nm, corresponding to reduced [Ru]˙^+^ and solvated electron (e_solv_), respectively.^58–60^ Notably, these species are the common photolyzed intermediates that originate in an aqueous environment and lead to decomposition.^58^ Interestingly, when the spectra were acquired in the presence of [Mn] under Ar, the absorption band centred at 550 nm increased, accompanied by a decrease in GS bleach. This clearly indicates an increase in the formation of a reduced PS ([Ru]˙^+^), confirming electron transfer to the PS, *i.e.*, the excited state of [Ru] is reductively quenched. It is further notable that when both systems ([Ru] alone and [Ru] + [Mn]) were purged with air, a decrease in the absorption band in this region was observed. This effect was more pronounced in the [Ru] + [Mn] system, irrespective of the sequence of addition of [Mn] and air. These results suggest that O_2_ acts as an electron acceptor, reverting the reduced PS to its ground state. This observation not only supports the validity of the initial reductive quenching mechanism but also illustrates the second step of the electron transfer process occurring between light-driven and dark reactions. Collectively, the results indicate that the PS is quenched by [Mn] *via* a reductive quenching mechanism, with no clear evidence of oxidative quenching.

Additionally, in the stimulated ^3^MLCT emission (SE) region around 660 nm,^61^ a significant negative bleaching effect was observed under aerobic conditions, more prominently in the [Ru] + [Mn] system, supporting the partial energy transfer mechanism as well in the photocatalytic systems.

Electrochemical study and ns-transient absorption spectroscopy confirm that electron transfer thermodynamics are evident under homogeneous conditions. These homogeneous studies provide essential reference points for interpreting the corresponding behaviour of the components in the MOF framework. The integration of Ru- and Mn-complexes into a unified UiO-67 framework (UiO-67 Ru_50_-Mn_10_) retains this synergistic character, as shown by phosphorescence quenching experiments (Fig. 8c, d and Table 4) by comparing between UiO-67 Ru_50_ and UiO-67 Ru_50_-Mn_10_. However, a comparison between homogeneous *vs.* MOF systems clearly highlights the contribution of the MOF (Table 4). The spatial proximity of Ru and Mn within the framework facilitates more efficient electron transfer than in homogeneous [Ru] + [Mn] systems, while the rigid architecture of the MOF enhances the stability of the photoactive units compared to homogeneous conditions, particularly in a CH_3_CN/H_2_O.

Although the ^3^MLCT states of Ru-complexes are not energetically suitable for O_2_˙^−^ formation,^39^ they can undergo energy transfer with ambient ^3^O_2_ to produce ^1^O_2_.^62^ This EnT mechanism is more prominent in CH_3_CN and homogeneous systems than in MOFs.^40^

**Fig. 11:**
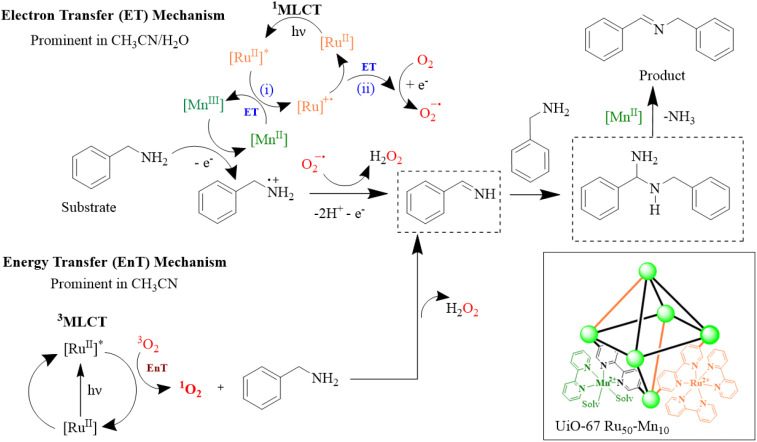
Oxidative amine coupling reaction mechanisms through the electron transfer (ET) and energy transfer (EnT) pathways.

Spectroscopic and photocatalytic results and intermediate identification across solvent environments suggest both electron transfer (ET) and energy transfer (EnT) pathways can operate in UiO-67 Ru_50_-Mn_10_ to convert benzylamine and O_2_ into *N*-benzylidene-1-phenylmethanamine (Fig. 11). In the ET pathway (Fig. 11 and S14–S21): photoexcitation converts [Ru^II^] to [Ru^II^]* (^1^MLCT), which is reduced by [Mn^II^] to generate [Ru]˙^+^ and becomes [Mn^III^]. O_2_ accepts an electron to [Ru]˙^+^ to regenerate [Ru^II^], by becoming O_2_˙^−^. [Mn^III^] (*E*° = +1.10 V *vs.* NHE) oxidizes benzylamine (*E*° = +0.76 V *vs.* NHE) to benzylamine radical (PhCH_2_NH_2_˙^+^), which reacts with O_2_˙^−^ to form phenylmethanimine (Im_1_) and H_2_O_2_. In the EnT pathway (Fig. 11, bottom): ^3^O_2_ formed *via*^3^MLCT of [Ru^II^]* reacts with benzylamine to yield the same intermediates. Formation of H_2_O_2_ through ^1^O_2_ and O_2_˙^−^ is well established and was also confirmed for the UiO-67 Ru_50_-Mn_10_ MOF using the TiOSO_4_ method^63,64^ (see SI and Fig. S29). Finally, phenylmethanimine, formed *via* both pathways, undergoes a condensation reaction to generate the hemiaminal intermediate (Im_2_), which subsequently eliminates NH_3_ to yield the final product. It is hypothesised that the rapid decay of intermediates in pure CH_3_CN prevents their detection in both the UiO-67 Ru_50_-Mn_10_ MOF and the homogeneous systems. In contrast, in CH_3_CN/H_2_O the decay of intermediates is relatively slower, enabling their observation (Fig. 6 and S14–S21).

UiO-67 Ru_50_-Mn_10_ exhibits significantly enhanced photocatalytic performance, showing ∼3.1, 1.65, and ∼1.8-fold higher TON than [Ru] alone, [Ru] + [Mn], and UiO-67 Ru_50_, respectively, in CH_3_CN/H_2_O. The superior photocatalytic activity of UiO-67 Ru_50_-Mn_10_ over UiO-67 Ru_50_, homogenous [Ru] alone, and [Ru] + [Mn] systems in CH_3_CN/H_2_O reflects a prominent role of the ET pathways and the role of Mn in facilitating ET for the primary one-electron oxidation of benzylamine. This role of manganese as an electron transfer mediator was previously reported.^65^

While the homogeneous system in CH_3_CN shows superior activity than MOFs due to a more efficient EnT *via*^3^MLCT, Mn remains critical in both solvent systems for promoting oxidation, thereby enhancing overall reaction kinetics in both [Ru] + [Mn] and UiO-67 Ru_50_-Mn_10_ compared to systems without Mn.

## Conclusion

This study demonstrates that incorporating Mn^2+^ into a Ru(bpy)_3_^2+^-functionalized MOF (UiO-67 Ru_50_-Mn_10_) significantly enhances the photocatalytic oxidation of benzylamine to *N*-benzylidene-1-phenylmethanamine by O_2_ from the air in CH_3_CN/H_2_O, achieving a TON of 634, approximately 1.65 times higher than the homogeneous system and 1.8 times higher than the Ru-only MOF (UiO-67 Ru_50_). Mechanistic investigations reveal the coexistence of electron transfer (ET) and energy transfer (EnT) pathways, with their relative contributions strongly influenced by the solvent environment. The CH_3_CN/H_2_O (4 : 1) solvent system plays a critical role by improving the polarity of the reaction medium, which collectively facilitates faster and more efficient charge separation and electron transfer. While homogeneous systems benefit more in pure CH_3_CN due to faster energy transfer (EnT), the MOF-based catalyst performs best in the mixed solvent due to stabilized intermediates and enhanced electron transfer pathways (ET). Spectroscopic and electrochemical studies further confirm enhanced photostability and charge dynamics in the MOF system. The MOF system provides all advantages of MOFs, being a dispersible, highly porous materials that maintains the molecular functions of its building blocks and has the potential for easy recyclability. The cooperative interaction between Ru and Mn centres within the robust MOF framework provides a blueprint for designing efficient, photocatalysts using earth-abundant catalysts for sustainable oxidative chemistry.

## Experimental section

### Synthesis of UiO-67

The zirconium-based metal–organic framework (UiO-67) was synthesized *via* a solvothermal method with slight modifications from previously reported procedures.^66^ ZrCl_4_ (260 mg, 1.12 mmol) and benzoic acid (6.8 g, 56 mmol, 50 equiv.) were added to 50 mL of *N*,*N*-dimethylformamide (DMF) in a 100 mL round-bottom flask and sonicated for 10 minutes to ensure uniform dispersion and partial dissolution of the precursors. In a separate vessel, 2,2′-bipyridine-5,5′-dicarboxylic acid (H_2_bpydc, 282 mg, 1.16 mmol) was suspended in 20 mL of DMF. Due to limited solubility, triethylamine (up to 320 μL) was added incrementally (20 μL per addition) under stirring until a clear solution was achieved. The linker solution was then slowly added to the ZrCl_4_–benzoic acid mixture with continuous stirring. After 10 minutes of additional mixing, the complete reaction solution was transferred to a 100 mL Teflon-lined stainless-steel autoclave and heated at 120 °C for 72 hours under static conditions. After cooling to room temperature, the resulting crystalline solid was collected by centrifugation at 4440 rpm and washed with fresh DMF (40 mL), followed by a second centrifugation. The solid was soaked in ethanol (40 mL) overnight to promote solvent exchange, and then washed three additional times each with ethanol and acetone to remove residual modulators and solvents. Each washing step was followed by centrifugation at 4400 rpm. The purified product was dried under vacuum and further dried at 60 °C in an oven before characterization or further use. The final yield was 298.4 mg, corresponding to a 75% yield based on ZrCl_4_ (theoretical yield: 398.1 mg).

### Synthesis of Ru(bpy)_3_^2+^-functionalized MOF (UiO-67 Ru_10_ and UiO-67 Ru_50_)

With a slight modification reported from the literature^66^ 65 mg of ZrCl_4_ (0.28 mmol) and 1.7 g of benzoic acid (14 mmol, 50 mol equiv.) were dissolved by sonication (5 min) in 10 mL of DMF. This was then added to a vial containing (1) 63.74 mg of H_2_bpydc (0.261 mmol) and 21.12 mg of [Ru(bpy)_2_(bpydc)]Cl_2_ (0.029 mmol) for UiO-67 Ru_10_ or (2) 35.41 mg of H_2_bpydc (0.145 mmol) and 105.64 mg of [Ru(bpy)_2_(bpydc)]Cl_2_ (0.145 mmol) for UiO-67 Ru_50_. As mentioned earlier, triethylamine (∼80 μL) was added incrementally (20 μL per addition) under stirring until a clear solution was achieved before the mixing with the ZrCl_4_–benzoic acid mixture. The solution was sonicated for an additional 5 min and transferred to a 100 mL Teflon-lined stainless-steel autoclave and heated at 120 °C for 72 hours and followed by a similar procedure used for pristine UiO-67.

#### Post-synthetic modification

##### Synthesis of Mn^2+^-doped UiO-67 Ru_*x*_ (UiO-67 Ru_10_-Mn_10_/UiO-67 Ru_50_-Mn_10_)

UiO-67 Ru_10_ or UiO-67 Ru_50_ (60 mg) was suspended in 10 mL of ethanol in a round-bottom flask, followed by the addition of 0.015 or 0.05 mmol of 1 : 1 equiv. of MnCl_2_·4H_2_O and 2,2′-bipyridine (bpy). The resulting suspension was stirred under an argon atmosphere at reflux temperature for 4 h to facilitate coordination of Mn(ii) to the Ru-functionalized MOF framework. Upon completion, the solid product was isolated by centrifugation, thoroughly washed with ethanol to remove any unbound metal species or ligands, and dried under vacuum before use.

##### Synthesis of Mn^2+^-doped UiO-67 (UiO-67 Mn_10_)

UiO-67 Mn_10_ was synthesized by following the same procedure described for UiO-67-Ru metalation, using pristine UiO-67 (60 mg) in place of UiO-67 Ru_10_ under identical conditions.

#### Photocatalytic oxidation of benzylamine

In a typical procedure, 1 mg of MOF powder was dispersed in 4 mL of a CH_3_CN : H_2_O (4 : 1 v/v) solvent mixture in an 8 mL vial and ultrasonicated for 5 minutes to ensure homogeneous dispersion. Subsequently, 0.734 mmol of benzylamine (80 μL) was added using a micropipette. The reaction mixture was kept in the dark overnight at ambient conditions. After the dark period, the vial was irradiated using a 460 nm LED light source (Azula Photoreactor, *λ* = 460 nm, 800–1250 mW) operating at 0.7 A and 4 V under continuous stirring.

For control experiments, the same procedure was followed by replacing the MOF catalyst with either (1) a combination of 0.28 μmol of [Ru(bpydc)(bpy)_2_]Cl_2_ and 0.14 μmol of [Mn(bpy)_2_Cl_2_]H_2_O, or (2) 0.28 μmol of [Ru(bpydc)(bpy)_2_]Cl_2_ (0.204 mg) alone in 4 mL of CH_3_CN with 0.734 mmol of benzylamine (80 μL).

At selected time intervals (initial, 0 h (dark), 1–24 h for kinetics; and 24 h for final yield), 240 μL aliquots were withdrawn and centrifuged at 15 000 rpm for 5 minutes to remove the solid MOF. Then, 200 μL of the supernatant was transferred to a rotavapor vial, and CH_3_CN was evaporated under reduced pressure at 50 °C, 150 rpm, gradually lowering the pressure from 230 mbar to 72 mbar, followed by full vacuum. The residue was redissolved in 800 μL of CD_3_CN containing a known quantity of internal standard (1,3,5-trioxane, 3 mg, 0.037 mmol), transferred to an NMR tube, and analysed by ^1^H NMR (64 scans).

For homogeneous control reactions, the centrifugation step was omitted. After evaporation of the solvent from 200 μL of the reaction mixture, the residue was treated with 800 μL of CD_3_CN containing the internal standard and analysed directly by ^1^H NMR.

#### Cyclic voltammetry

Cyclic voltammetry (CV) experiments were performed on a Pine Research Wavedriver 200 electrochemical workstation equipped with a standard three-electrode arrangement: working electrode (WE): glassy carbon electrode (*d* = 3.0 mm), quasi-reference electrode (RE): Ag/AgCl, counter electrode (CE): Pt wire. All potentials are quoted relative to the ferrocene/ferrocenium internal standard. All experiments were performed in dry acetonitrile (CH_3_CN) using *n*Bu_4_NPF_6_ (0.1 M) as supporting electrolyte. The solutions were purged with argon for at least 15 minutes to remove O_2_ and kept under a slight positive argon pressure when performing the experiments.

## Conflicts of interest

There are no conflicts to declare.

## Supplementary Material

RA-015-D5RA04503G-s001

## Data Availability

The data supporting the findings of the article including detailed experimental descriptions can be found in the supplementary information (SI). Supplementary information is available. See DOI: https://doi.org/10.1039/d5ra04503g.
