# Supplementary material for: Mn2+ and [Ru(bpy)3]2+ in UiO-67 metal organic frameworks enhance photocatalytic oxidation of benzylamine via an electron transfer pathway
Source: RSC Adv. 2025 Oct 13;15(45):38105–21. doi: 10.1039/d5ra04503g (PMC12517270; doi:10.1039/d5ra04503g)
Supplement: RA-015-D5RA04503G-s001 [file RA-015-D5RA04503G-s001.pdf]

Supplementary Information (SI) for RSC Advances.
This journal is © The Royal Society of Chemistry 2025

Mn^{2+} and $[Ru(bpy)_3]^{2+}$ in UiO-67 Metal Organic Frameworks Enhance Photocatalytic Oxidation of Benzylamine via Electron Transfer Pathway

Subrata Mandal,[a] Novitasari Sinambela,[a] Johannes Biskupek,[b] Riccarda Müller,[c] Ute Kaiser,[b] Kerstin Leopold,[c] and Andrea Pannwitz*[a, d, e, f]

[a] Institute of Inorganic Chemistry I, UniversityUlm, Albert-Einstein-Allee 11, 89081 Ulm, Germany

[b] Central Facility of Electron Microscopy, Electron Microscopy Group of Material Science, University of Ulm, Albert-Einstein-Allee 11, Ulm 89081, Germany

[c] Institute of Analytical and Bioanalytical Chemistry, University Ulm, 89081 Ulm, Germany

[d] Institut for Inorganic and Analytical Chemistry, Friedrich-Schiller-UniversiyJena, Humboldtstr. 8, 07743 Jena, Germany

[e] Center for Energy and Environmental Chemistry Jena (CEEC), Friedrich-Schiller-Universität Jena, Philosophenweg 7a, 07743 Jena

[f] Helmholtz Institute for Polymers in Energy Applications Jena (HIPOLE Jena), Lessingstraße 12–14, 07743 Jena, Germany

Contents

Experimental Section 2
Materials and Reagents 2
Chemicals: 2
Synthesis of $[Ru(bpy)_2(bpydc)]Cl_2$ 2
Synthesis of $[Mn(bpy)_2Cl_2]H_2O$: 3
Instrumentations 3
Protocol for MOF Digestion and 1H NMR Analysis 10
Photocatalytic activity for the oxidation of benzylamine 14
Calculations of the percentage of yields and TON and TOF: 15
Spectroscopic studies 22
Cyclic voltammetry (CV) 26
Energy level of HOMO and LUMO in $[Ru(bpy)_2(bpydc)]Cl_2$, and reduction potential of $[Mn(bpy)_2Cl_2].H_2O$ and O_2 (*vs* NHE): 27
Quantification of H_2O_2 using the $TiOSO_4$ method 27
References 28

Experimental Section

Materials and Reagents

Chemicals: Zirconium tetrachloride ($ZrCl_4$, ≥99.5%), benzoic acid (≥ 99.5%), benzylamine (99%), cis-dichlorobis (2,2′-bipyridine) ruthenium (II) hydrate ($Ru(bpy)_2Cl_2$. xH_2O, 97%), manganese (II) chloride tetrahydrate ($MnCl_2.4H_2O$) were purchased from Sigma Aldrich. Dry N, N-dimethylformamide (DMF) and acetonitrile (CH_3CN) solvents were used as received. Unless otherwise stated, any other chemicals (99.9% purity or higher) were used without further purification.

Synthesis of $[Ru(bpy)_2(bpydc)]Cl_2$: [1] In 20 mL of water and ethanol (1:1), 0.42 mmol (101 mg) of H_2bpydc and 0.33 mmol (160 mg) of $[Ru(bpy)_2Cl_2]$ are mixed and then heated in an Ar atmosphere for 9 hours. After the reaction, the solution was cooled down, and the solvent was removed under low pressure by rotavapor. The solids obtained were re-dissolved in Et_2O/MeOH and kept for recrystallization. The precipitate was filtered and left to dry in an oven at 40°C overnight. The red solid $[Ru(bpy)_2(bpydc)]Cl_2$ is characterized by ^{1}H NMR and UV-vis absorption spectroscopy. ^{1}H NMR (D_2O): δ 8.52 (d, 2H); 8.44 (m, 4H); 8.27 (d, 2H);

8.10 (s, 2H); 7.96 (q, 4H); 7.74 (m, 4H); 7.29 (dd, 4H). (Yield 65%). UV-vis (CH_3CN): λ_{max}, ^{1}MLCT at 460 nm

Synthesis of [Mn(bpy)$_2$Cl$_2$]H$_2$O: This complex was prepared as per the reported procedure.[2–4] Briefly, to a solution of 2,2′-bipyridyl (bpy) (0.984 g, 6.3 mmol) in ethanol (30 mL), $MnCl_2 \cdot H_2O$ (0.363 g, 2.5 mmol) was added, and the mixture was warmed gently and stirred for 1.5 h at refluxing conditions. Then, the yellow precipitate obtained was filtered off, washed with small amounts of cold methanol, and dried under vacuum. The crude product was further dissolved in a water/ethanol mixture for crystallization to achieve [Mn(bpy)$_2$Cl$_2$]H$_2$O.

Instrumentations

^{1}H nuclear magnetic resonance (NMR) spectroscopy for the synthesized [Ru(bpy)$_2$(bpydc)]Cl$_2$ and digested UiO-67-based MOFs was performed at 298 K using a Bruker DRX 400 MHz spectrometer. Chemical shift values are reported in parts per million (ppm) and referenced to the residual peaks of the corresponding solvent. The spectra were analyzed using MestReNova software. Infrared spectra were recorded using a Bruker Alpha II spectrometer equipped with an ATR Platinum Diamond unit. The data were collected over 24 scans at a resolution of 4 cm^{-1}. A PANalytical X'Pert PRO diffractometer, equipped with Cu Kα radiation and a Pixel detector, was employed to acquire powder X-ray diffraction (PXRD) patterns of the MOF samples. Data were collected over a 2θ range of 5–20° at a scan rate of 0.5° min^{-1}.
TEM analysis for the MOFs was carried out with a Jeol 1400 TEM. For sample preparation, the MOF particles were first dispersed in ethanol via ultrasonication. The resulting dispersion was then drop-casted on a copper TEM support grid with holey carbon film and allowed to dry before analysis. High-resolution transmission electron microscopy (HRTEM) as well as Scanning transmission electron microscopy (STEM), in combination with energy-dispersive X-ray spectroscopy (EDX), was conducted on the MOF particles using a ThermoFisher Talos F200X microscope. This instrument was operated at an acceleration voltage of 200 kV, dedicated sample holder made of beryllium was used together with the embedded SuperX windowless EDX detector to collect X-rays with maximum efficiency. A similar sample preparation method is followed as stated earlier. Quantification of EDX data (mappings, spectra, elemental ratios) was determined using the Velox software package (Thermofisher Scientific company), applying background subtraction and Schreiber-Wims kFactor quantification.
Diffuse-reflectance UV-vis spectra of the MOF solids were recorded using a Shimadzu UV-2600 UV-vis spectrophotometer. The measurements were performed on samples mixed with $BaSO_4$ as a reference. X-ray photoelectron spectroscopy (XPS) measurements were conducted by placing the powdered samples onto a conductive Au substrate, which was then secured to the XPS sample holder. Data acquisition was performed using a UHV Multiprobe system (ScientaOmicron, Germany) equipped with a monochromatic Al Kα X-ray source and an Argus CU electron analyzer, offering an energy resolution of 0.6 eV. Charge compensation was achieved using an electron flood gun (NEK 150, Staib, Germany) operating at 6 eV and 50 µA. Background subtraction was applied, and the spectra were calibrated to the C 1s peak at 284.6 eV before fitting with Voigt functions (30:70 ratio).

For total X-ray fluorescence spectrometry (TXRF) measurements high-efficiency module S2 Picofox (Bruker Nano GmbH, Berlin, Germany) equipped with Mo X-ray tube was used. The measurement live time was set to 1000 s and excitation of the sample was carried out at maximum power conditions (50 kV and 600 µA). For the quantification of Mn and Ru the samples were digested using 1 mL conc. HNO_3. Ga standard solution (100 mg L^{-1} in 2 wt.-% HNO_3, Honeywell Fluka, USA) was added as internal standard to 500 µL of the sample. After homogenization, 10 µL of each sample were pipetted onto pre-cleaned and siliconized quartz glass sample carriers and dried for 90 min at 60 °C. All samples are prepared in triplicate. Spectra PicoFox (7.2.5.0, Bruker Nano GmbH) software was used to evaluate the obtained spectra, and for deconvolution, the profile bayes normal fit was selected. Ruthenium is difficult to evaluate due to overlap of the Ru L_{α} (2.56 keV) signal with the chlorine K_{α} (2.62 keV) signal.

Therefore, quantification of Ru was carried out using high resolution-continuum source-graphite furnace atomic absorption (HR-CS-GFAAS) spectrometer ContrAA 600 (Analytik Jena GmbH, Germany) equipped with a graphite furnace atomization unit and XK3BC3051 auto sampler for liquid sampling (Analytik Jena GmbH, Germany). Argon with a purity of 99.996% (MTI, Neu-Ulm, Germany) was used as purge and protective gas. The temperature program used for the quantification of Ru can be found in **Table S1**. For the measurements, the solutions prepared for TXRF analysis were used after appropriate dilution by 5% HCl. Each sample was measured in triplicate, and 20 uL were pipetted into the furnace tube. The most sensitive line of Ru (349.895 nm) was used and quantification was done by external calibration using Ru stock standard solution (20 µg L^{-1}) diluted from 1000 mg L^{-1} standard solution (in 5 wt.-% HCl, VWR international, Belgium).

Table S1: Used temperature program for the Ru determination using HR-CS-GF-AAS.

Step	Name	Temperature / °C	Ramp / °C s^{-1}	Hold / s	Time / s	Gas int.
1	Drying	85	6	35	44.2	Max
2	Drying	95	1	40	50.0	Max
3	Drying	110	1	45	60.0	Max
4	Pyrolysis	550	15	15	44.3	Max
5	Pyrolysis	1000	300	15	16.5	Max
6	Gas adjustment	1000	0	5	5.0	Stopp
7	Atomization	2700	1500	6	7.1	Stopp
8	Clean out	2750	500	4	4.1	Max

Unless otherwise stated, all spectroscopic samples were prepared in 1 cm pathlength airtight quartz glass cuvettes in an argon-filled glovebox (MBraun) and typically handled at 20 °C (room temperature). Solvents used for spectroscopic analyses were of HPLC grade or higher and were stored under argon in the glovebox. Steady-state UV-Vis absorption spectroscopy was performed on a V-760 JASCO UV-VIS-NIR Spectrophotometer, and steady-state emission spectroscopy was performed on a JASCO FP-8500 Spectrofluorometer.

Fluorescence lifetimes were recorded with a DeltaPro from Horiba Scientific using a 372 nm pulsed Laser source (Class 3B Laser Product, <0.5 W peak in pulsed and CW mode) and a 495 nm long pass filter. The Delta Pro consists of: DeltaDiode (Picosecond diode controller), DeltaHub (High throughput TCSPC controller), DPS-1 (Detector Power supply), and a PPD (Picosecond photon detection module). The Instrument response function (IRF) was measured with LUDOX silica nanoparticles. Data fitting was performed using Origin software.

Transient absorption experiments occurred on an LP980-K spectrometer from Edinburgh Instruments equipped with an iCCD detector from Andor (DH320T-25 F-03-812), a monochromator (STGM325-MA), and a photomultiplier (PMT-LP R928P). The excitation source (pump) utilizes a pulse Nd: YAG/YVO4 laser from Ekspla (NT342B-10-AW) equipped with a tunable OPO (410-2600 nm) and with a repetition rate of 10 Hz. The probe light running at pulsed mode (10 Hz) was generated by a 150W ozone-free xenon arc lamp with Spectrometer Controller (LP1). The sample chamber was tempered to 20 °C using a built-in thermostat. For all spectral measurements, 20 pulses were averaged, and the signal was integrated over a 100 ns timespan to yield the data shown. The signal of 20 pulses was averaged to improve the signal-to-noise ratio, and for each pulse, a gate width of 100 ns was chosen.

Cyclic voltammetry (CV) experiments were performed on a Pine Research Wavedriver 200 electrochemical workstation equipped with a standard three-electrode arrangement: working electrode (WE): glassy carbon electrode (d = 3.0 mm), quasi-reference electrode (RE): Ag/AgCl, Counter electrode (CE): Pt wire. All potentials are quoted relative to the ferrocene/ferrocenium internal standard. All experiments were performed in dry CH_3CN using nBu_4NPF_6 (0.1 M) as supporting electrolyte. The solutions were purged with argon for at least 15 minutes to remove O_2 and kept under a slight positive argon pressure while performing the experiments.

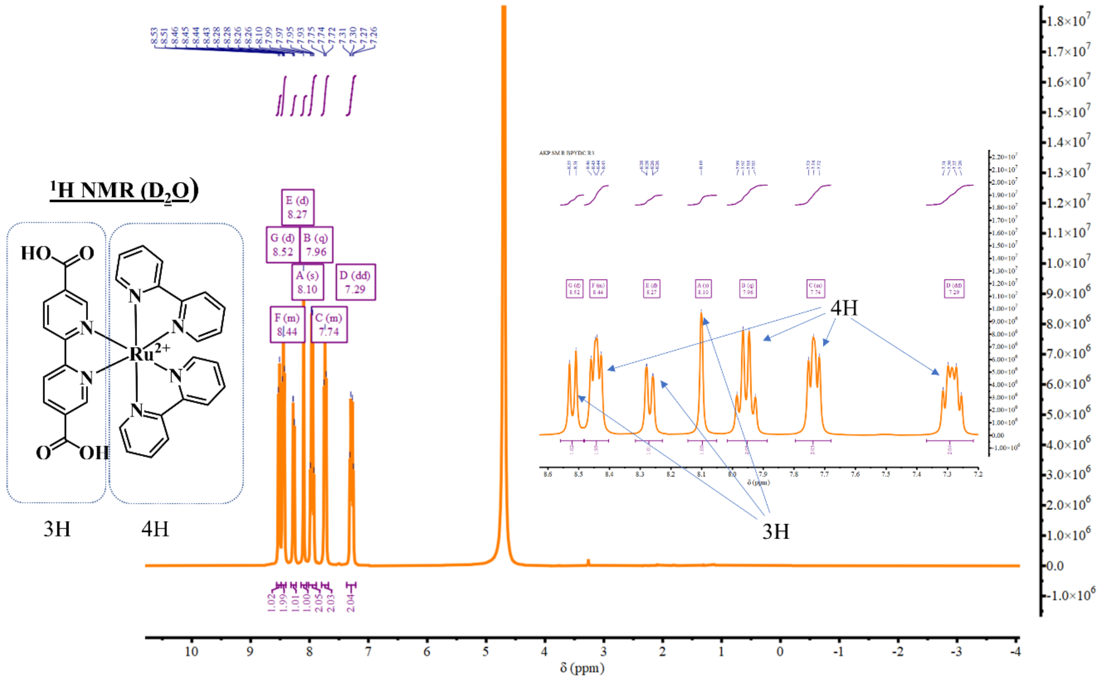

Figure S1. ^{1}H NMR spectra of the synthesized $[Ru(bpy)_2(bpydc)]Cl_2$ in D_2O. (400 MHz)

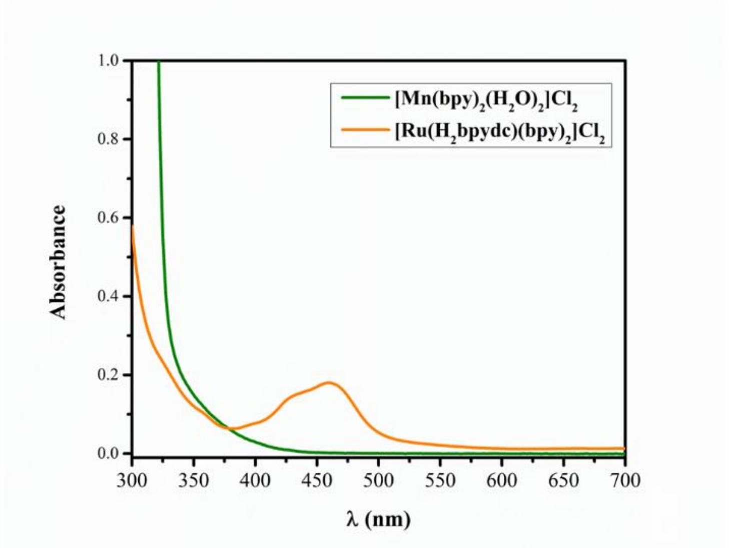

Figure S2. (a) Steady-state UV-vis absorption spectra of the synthesized $[Ru(bpy)_2(bpydc)]Cl_2$ (orange) (green) and $[Mn(bpy)_2(H_2O)_2]Cl_2$ in CH_3CN.

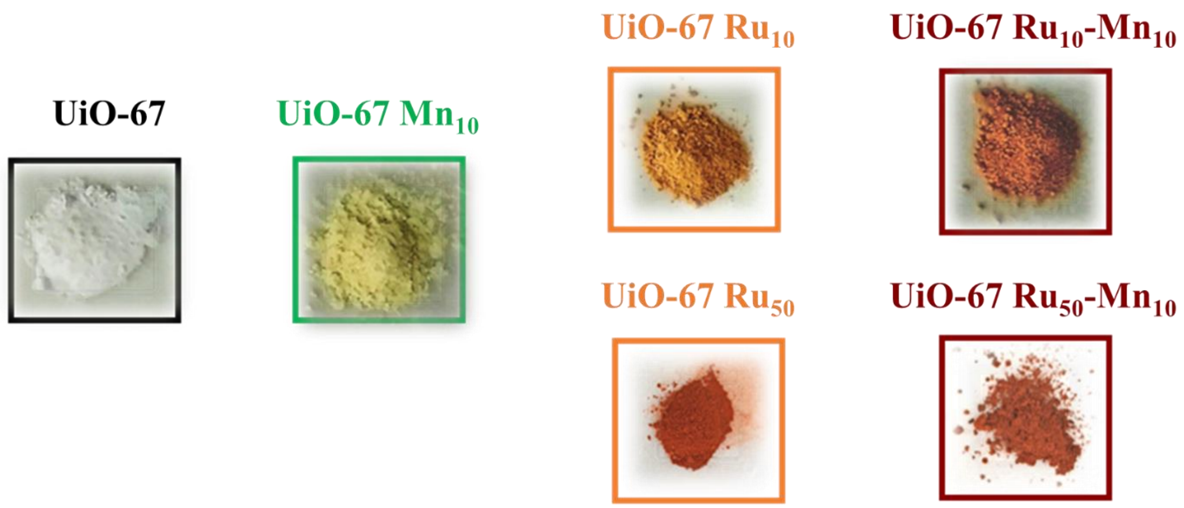

Figure S3. Photograph of the prepared samples.

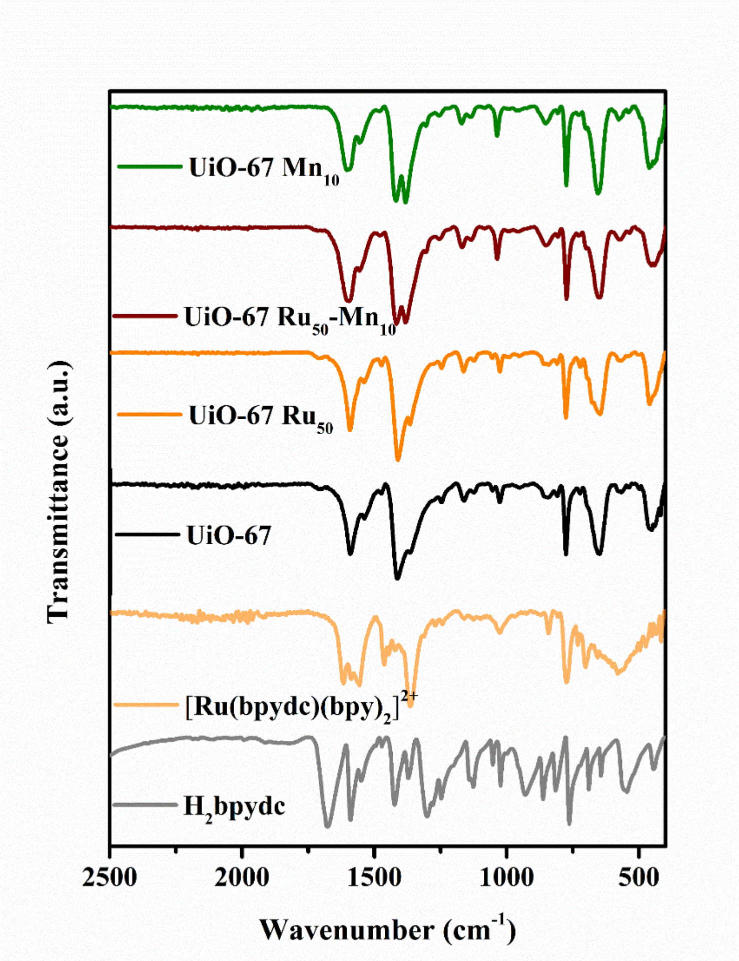

Figure S4. ATR -IR spectra of the prepared MOF samples and comparison with the free H_2bpydc and $[Ru(bpydc)(bpy)_2]^{2+}$ linker.

The ATR-IR spectra presented in **Figure S4** reveal that the asymmetric vibrational absorption bands corresponding to the C=O (1665 cm^{-1}) and C–OH (1290 cm^{-1}) groups in free H_2bpydc

are both reduced and blue-shifted in the UiO-67 MOFs. In addition, a pronounced peak emerges around 1410 cm^{-1}, attributed to the symmetric stretching of the carboxylate (-COO) group, indicating coordination of the carboxyl functionality to Zr centers.[5] This coordination feature is consistently observed across all MOF samples: UiO-67 Ru_{50}, UiO-67 Ru_{50}-Mn_{10}, and UiO-67 Mn_{10}, with slight variations in band position and shape noted. The assignment of these vibrational transitions in UiO-67 and UiO-67 Ru-based MOFs has been previously reported and discussed in the literature.[6,7] However, distinguishing the characteristic IR signals of free bpy in $[Ru(bpydc)(bpy)_2]^{2+}$ and $[Mn(bpy)_2]^{2+}$ proved inconclusive, due to spectral overlap with the bpydc moiety of the MOF and the low molar ratio of these species. As a result, definitive conclusions regarding their local coordination environment within the framework could not be drawn.

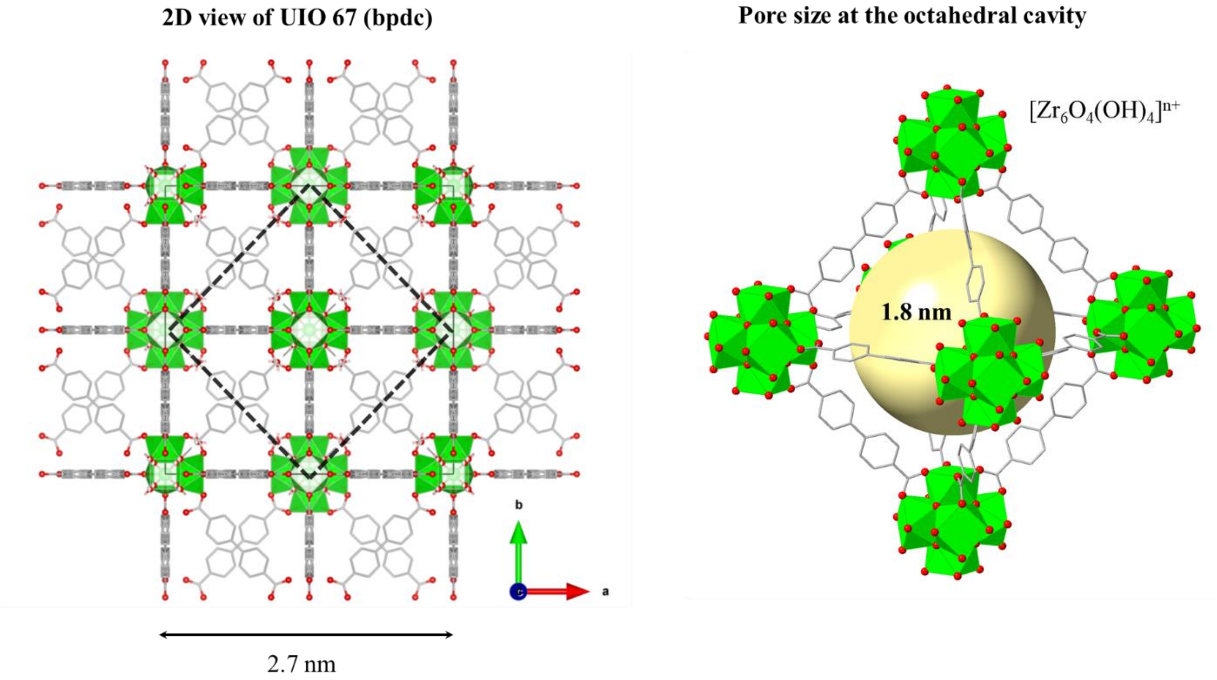

Figure S5. The eight triangular faces of the octahedron are alternately capped by four μ_3-hydroxo (μ_3-OH) and four μ_3-oxo (μ_3-O) groups. Each Zr(IV) centre demonstrates eight-coordination with oxygen atoms, adopting a square antiprismatic coordination environment. Notably, one square face of this antiprism is formed by four oxygen atoms originating from carboxylate groups, while the opposing square face is comprised of four oxygen atoms, two each from the μ_3-OH and μ_3-O groups. Inorganic octahedral Zr_6 units connect to twelve other similar units through biphenyl dicarboxylate linkers. This creates accessible microporous cages with supertetrahedral (~12 Å) and superoctahedral (~16 Å) cavities accessible through triangular windows. [8]

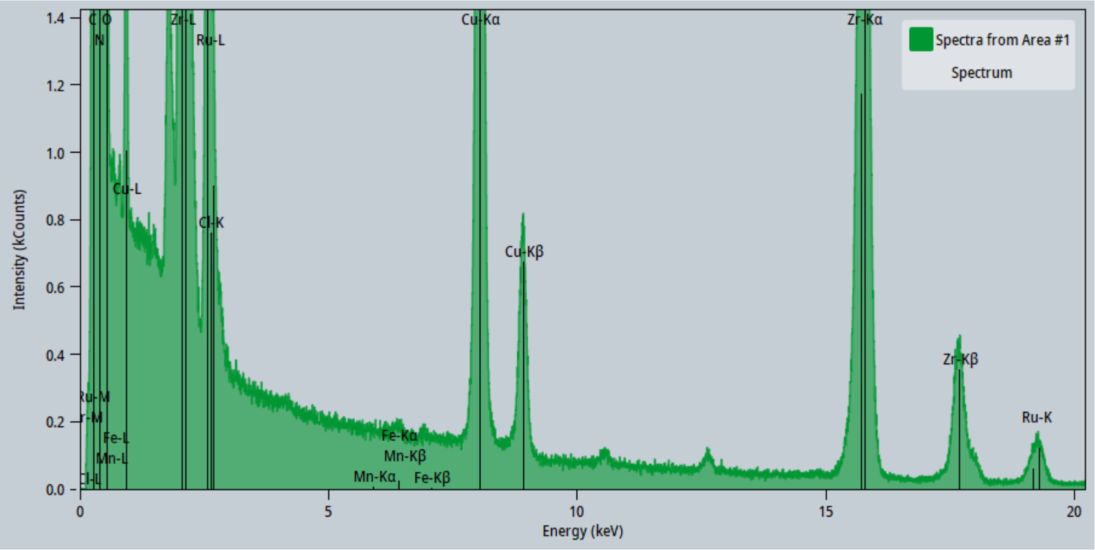

Figure S6. EDX spectrum of the UiO-67 Ru_{50}-Mn_{10} sample obtained from the HAADF-STEM image presented in Figure 3 of the main manuscript.

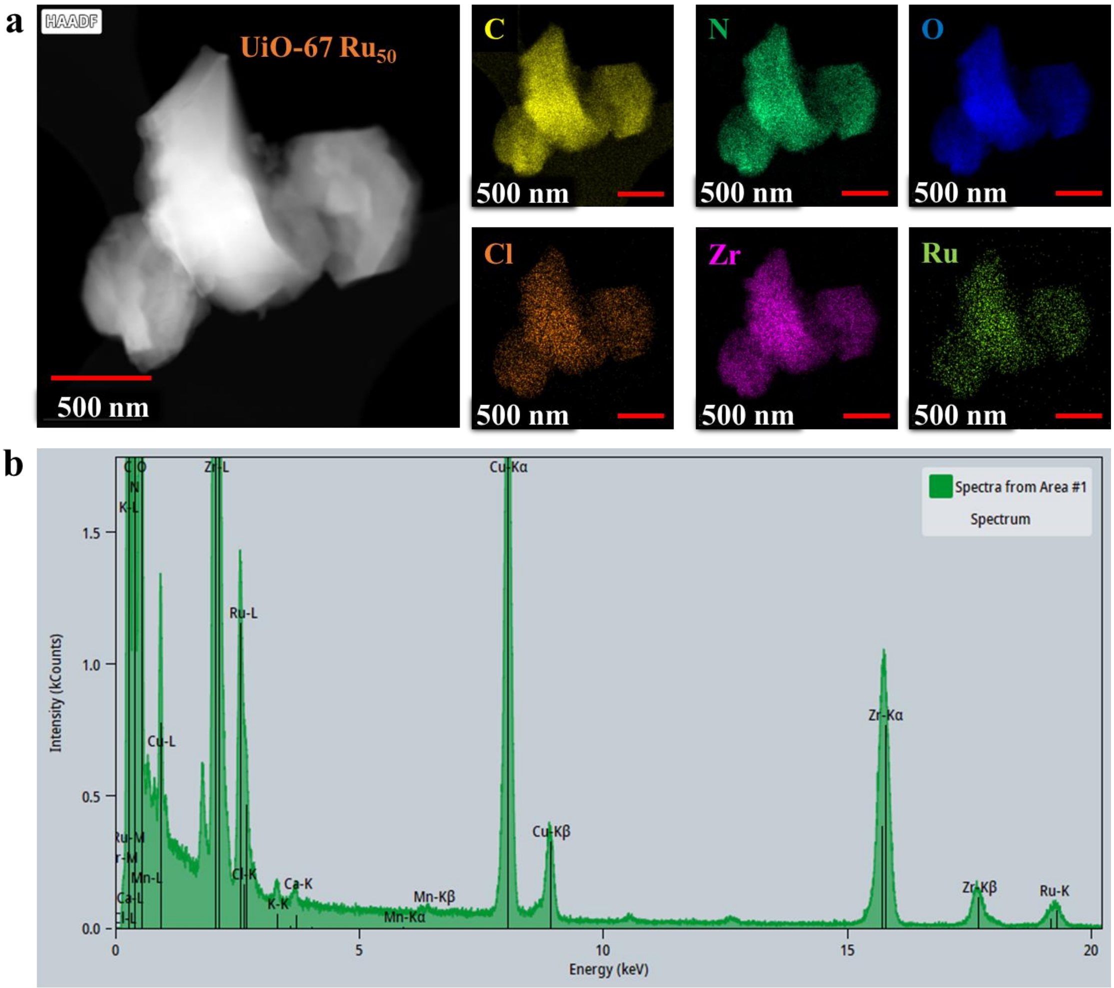

Figure S7. (a) HAADF-STEM image with corresponding EDX elemental mapping images and integrated (b) EDX spectrum of UiO-67 Ru_{50} sample. The yellow, dark green, blue, orange, purple, and light green signals represent C, N, O, Cl, Zr, and Ru, respectively.

Table S2. Elemental composition of the MOFs obtained from the EDX data in Figure S6- S7 (atomic%).

Samples	Atomic %							Ratio obtained from EDX	
	Zr	Ru	Mn	C	N	O	Cl	Zr/Ru	Expected ratio [b]
UiO-67 Ru_{50}	8.9±1.5	1.4±0.3	-	51.1±5.9	10.1±2.2	28.2±5.0	0.22±0.05	~6.35	2
	0.18±0.04	0.16±0.03	-	54±5.4	8.7±1.8	27.2±54.8	0.25±0.05	~1.12	
UiO-67 Ru_{50}-Mn_{10}	19.4±2.2	1.7±0.3	0.04[a]	46.5±2.7	5.9±1.2	25.8±3.9	0.6±0.1	~11.4	
	10.3±1.3	0.9±0.2	0.03[a]	54.9±3.1	7.3±1.4	26.2±3.9	0.4±0.1	~11.4	

[a] No detectable Mn in the spectrum above the noise level of about 0.1%, Mn-map is mostly an artifact because of the overlay of Mn Kβ with Fe Kα line, Fe is a stray signal on the TEM column.[b] expected stoichiometric ratio of Zr/Ru according to the proposed formulas: UiO-67 Ru_{50} ($[Zr_6O_4(OH)_4(bpydc)_3]\cdot 3[Ru(bpy)_2(bpydc)]Cl_2$) and UiO-67 Ru_{50}-Mn_{10} ($[Zr_6O_4(OH)_4(bpydc)_{2.4}]\cdot 3[Ru(bpy)_2(bpydc)]Cl_2\cdot 0.60[Mn(bpy)(bpydc)Cl_2]$). Cu signals are artifacts and background scattering of stray electrons at the TEM support grid.

Protocol for MOF Digestion and 1H NMR Analysis

The preheated MOF (1.2 mg) was placed in a 4 mL glass vial to prepare the samples. Subsequently, 90 µL of DCl/D_2O under air and 600 µL of dimethyl sulfoxide-d_6 were added to the vial. The vials were sealed, sonicated for 15 minutes, and then left at room temperature overnight to produce a uniform, transparent solution. These solutions were then transferred into NMR tubes, and ^1H NMR spectra were recorded using a Bruker DRX 400 MHz spectrometer (**Figure S8**). The spectra were referenced to tetramethylsilane, using the residual dimethyl sulfoxide-d^5 signal at δ = 2.50, and normalized to the intensities of the bpydc resonances observed between 8.60 and 9.20 ppm (δ 9.12 (s, 2H), δ 8.71 (d, 2 H), δ 8.65 (d, 2H)). The peaks between 7.30 and 8.00 ppm are due to benzoate (BA), (δ 7.81 (d, 2H), δ 7.51 (t, 1H), and 7.40 (t, 2H)), and formate (HCOO), (δ 8.10 (s, H)) that were also present in as-synthesized UiO-67. Notably, the formate peak is negligible and ignored for the current analysis. In UiO-67 Ru_x MOF, the $[Ru(bpy)_2(bpydc)]Cl_2$ linker can be characterized by ^1H NMR, and analysis was possible because it remains intact under these MOF digestion conditions. The signals are identified at δ 8.80-8.90 (d, 2H), 8.35 (d, 2H) ppm for bpydc moiety and 8.07 (m, 4H) ppm for bpy moiety in $[Ru(bpy)_2(bpydc)]Cl_2$ linker. The rest of the proton signals for these two moieties at around 8.60-8.75 ppm and 7.60-8.10 ppm, however, overlapped with the signals of the free bpydc linker and residual D_2O. Integration of the any of these distinct proton resonances from $[Ru(bpy)_2(bpydc)]Cl_2$ linker confirmed the degree of Ru modification (%) in UiO-67 Ru_x MOF, while the degree of Mn modification in UiO-67 Ru_x-Mn_y is not possible, as the

$[Mn(bpy)(bpydc)]Cl_2$ linker is diamagnetic, resulting in a broad signal as observed in UiO-67 Ru_{50}-Mn_{10}

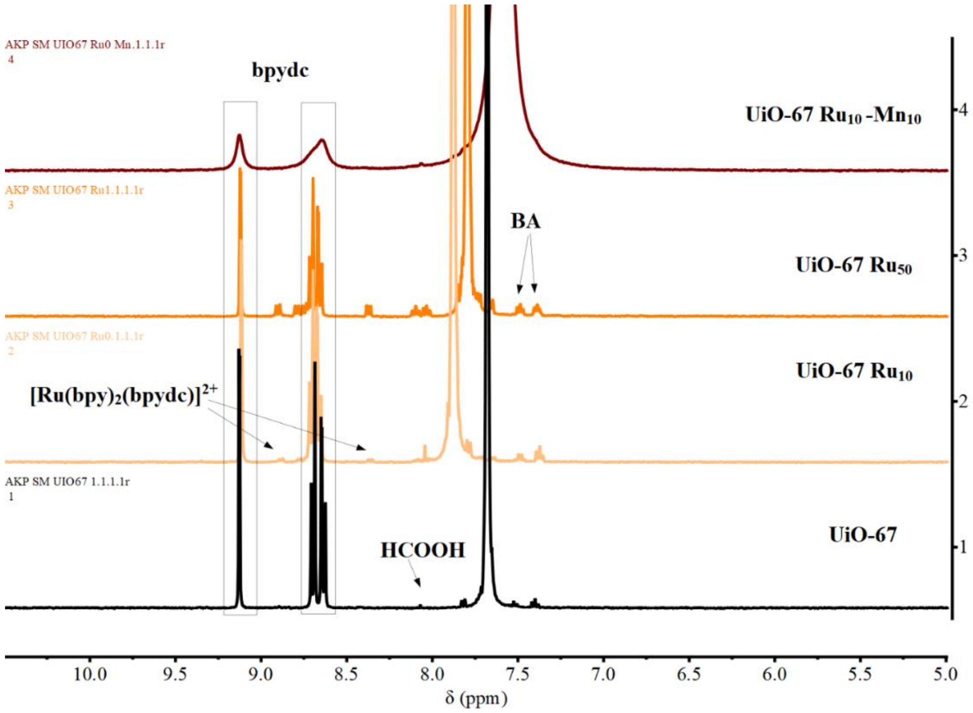

Figure S8. ^{1}H NMR spectra of the digested MOFs at different Ru content in UiO-67 Ru_x (UiO-67 Ru_{50} and UiO-67 Ru_{10}), and with different counterparts (UiO-67, and UiO-67 Ru_{10}-Mn_{10}).

The actual experimental molar ratio of the {BA}, {HCOO}, and $\{[Ru(bpy)_2(bpydc)]Cl_2\}$ to {bpydc} in UiO 67 was determined from **equation S1**

$$\frac{\{[\mathrm{Ru(bpy)2(bpydc)]Cl2}\}\ or\ \{\mathrm{BA}\}}{\{\mathrm{bpydc}\}} = \frac{\int\ [\mathrm{Ru(bpy)2(bpydc)]Cl2\ or\ BA}}{n_H\ ([\mathrm{Ru(bpy)2(bpydc)]Cl2\ or\ BA})} \times \frac{\mathrm{n_H\ (bpydc)}}{\int \mathrm{bpydc}} = \frac{n}{a}\ \mathrm{or}\ \frac{\mathrm{b}}{a} \qquad \text{(S1)}$$

, where $\int[\mathrm{Ru(bpy)2(bpydc)]Cl2\ or\ BA}$ is the integration of the $[Ru(bpy)_2(bpydc)]Cl_2$ or BA at δ ~8.80-8.90, and 7.40, respectively, $n_H([\mathrm{Ru(bpy)2(bpydc)]Cl2\ or\ BA})$ is the number of protons corresponding to the doublet (2H) and triplet (2H), respectively. n_H (bpydc) is the number of protons corresponding to the selected bpydc at 9.12 ppm, and $\int \mathrm{bpydc}$ is the integration of that bpydc singlet (2H).

The ideal chemical formula of the UiO 67 MOF is $Zr_6O_4(OH)_4(bpydc)_6$. Charge of the SBU: $[Zr_6O_4(OH)_4]^{12+}$, total charge of the 6-equiv. linker bpydc (2-) is 12. The obtained chemical formula from the experiments will be $[Zr_6O_4(OH)_4(bpydc)_a(BA)_b]$ in pristine UiO 67, where the SBU $[Zr_6O_4(OH)_4]^{12+}$ connects with a equiv. of bpydc, and b equiv. of BA. Based on the total charge,

$$\mathrm{b}+2a=12 \qquad \text{(S2)}$$

And for UiO-67 Ru_x, the total charge, $\mathrm{b} + 2(\mathrm{a}+\mathrm{n}) = 12$ (S3)

Therefore, using the molar ratio obtained from equation S1 and by solving the equation from equations S2 and S3, bpydc, BA, and $[Ru(bpy)_2(bpydc)]Cl_2$ can be calculated.

Table S3. Quantification of bpydc linker to $[Ru(bpy)_2(bpydc)]Cl_2$, BA, and HCOO in digested MOFs by ^{1}H NMR.

Samples	Relative area ^{1}H NMR			Molar ratio {bpydc}: {BA}:{$[Ru(bpy)_2(bpydc)]Cl_2$}	Calculated formula of the MOF $[Zr_6O_4(OH)_4(bpydc)_a (BA)_b]$ *n*[M]
	**bpydc** δ 9.12 (2H)	**BA** δ 7.40 (2H)	**$[Ru(bpy)_2(bpydc)]Cl_2$** δ 8.80-8.90 (2H)		
**UiO 67**	1	0.06	-	1:0.06	$[Zr_6O_4(OH)_4(bpydc)_{5.82}(BA)_{0.36}]$
**UiO 67 Ru_{50}**	1	0,24	0.15	1:0.24:0.15	$[Zr_6O_4(OH)_4(bpydc)_{4.724}(BA)_{1.13}]$ 0.70 $[Ru(bpy)_2(bpydc)]Cl_2$ (UiO-67 Ru_{13})
**UiO 67 Ru_{10}**	1	0.13	0.03	1:0.13:0.03	$[Zr_6O_4(OH)_4(bpydc)_{5.48}(BA)_{0.71}]$ 0.16 $[Ru(bpy)_2(bpydc)]Cl_2$ (UiO-67 Ru_3)
**UiO 67 Ru_{50}-Mn_{10}**			-[a]		$[Zr_6O_4(OH)_4(bpydc)_{3.944}(BA)_{1.13}]$ 0.70 $[Ru(bpy)_2(bpydc)]Cl_2$ 0.78 $[Mn(bpy)(bpydc)Cl_2]$ (UiO-67 Ru_{13}-Mn_{14})

[a] The ratio of Ru and Mn in UiO 67 Ru_{50}-Mn_{10} is considered from the combination of XPS data (Zr/Mn ratio) as listed in **Table S**4 and ^{1}H NMR data of UiO-67 Ru_{50} (Zr/Ru ratio) (assuming no leaching occurred during the post-synthetic modification, PSM).

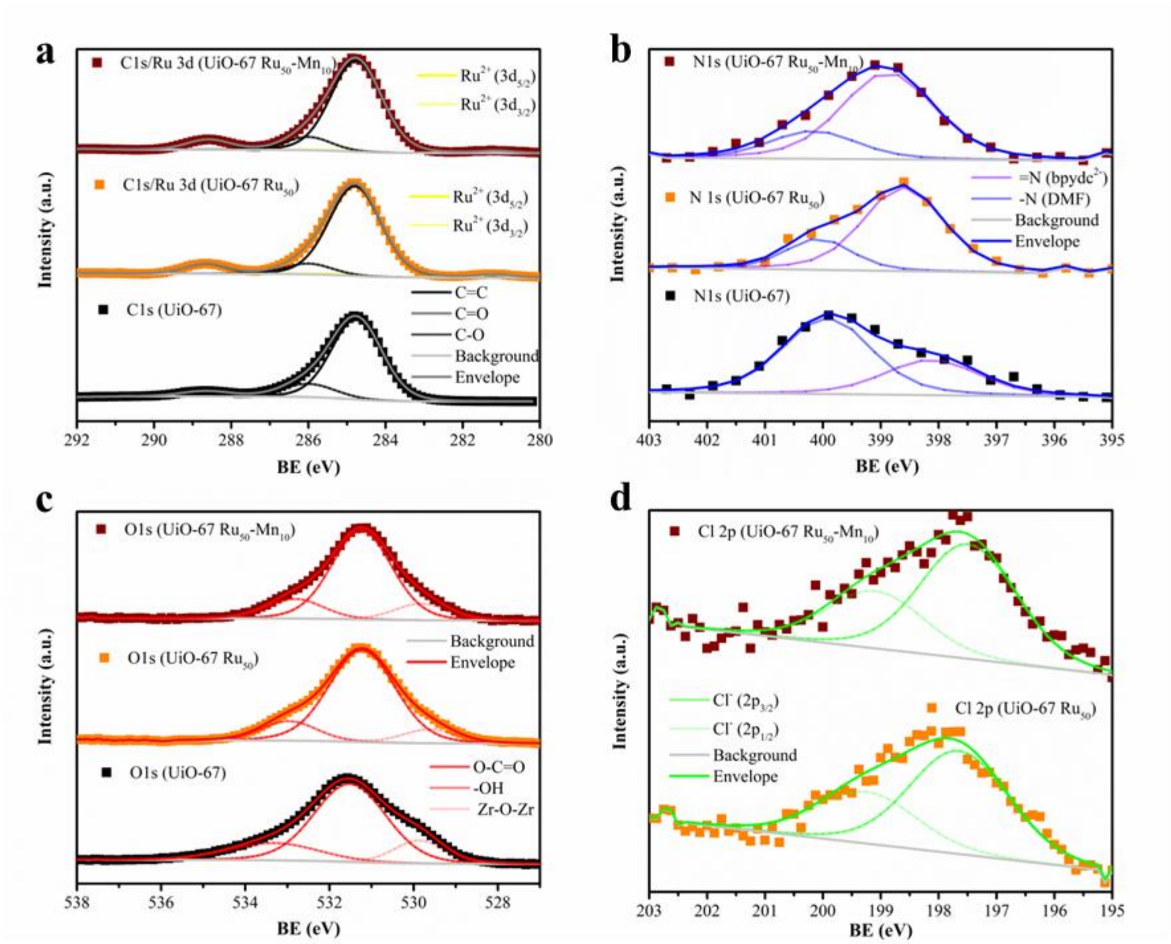

Figure S9. X-ray photoelectron spectra (XPS) of UiO-67-based materials: high-resolution (a) C 1s with overlapping Ru 3d, (b) N 1s, (c) O 1s spectra of UiO-67, UiO-67 Ru_{50}, and UiO-67 Ru_{50}-Mn_{10}, and (d) Cl 2p spectra for UiO-67 Ru_{50} and UiO-67 Ru_{50}-Mn_{10}.

Table S4. Chemical composition of the elements obtained from XPS analysis.

MOFs	Atomic (%) from XPS									NMR
	Zr	C	N	O	Ru[a]	Mn	Cl	Zr/Ru[a] ratio	Zr/Mn ratio	Zr/Ru
UiO-67	3.91	69.80	2.11	24.17	-	-	-	-	-	-
UiO-67 Ru_{50}	2.61	71.18	4.72	21.08	0.1	-	0.30	26.1	-	8.57
UiO-67 Ru_{50}-Mn_{10}	2.32	71.32	4.55	21.01	0.08	0.30	0.40	29	7.73	-

[a] Despite the presence of the Ru peak being detected in UiO-67 Ru-Mn, Ru was not taken into account for estimating the Ru/Mn ratio due to the overlap with C and a weak signal.

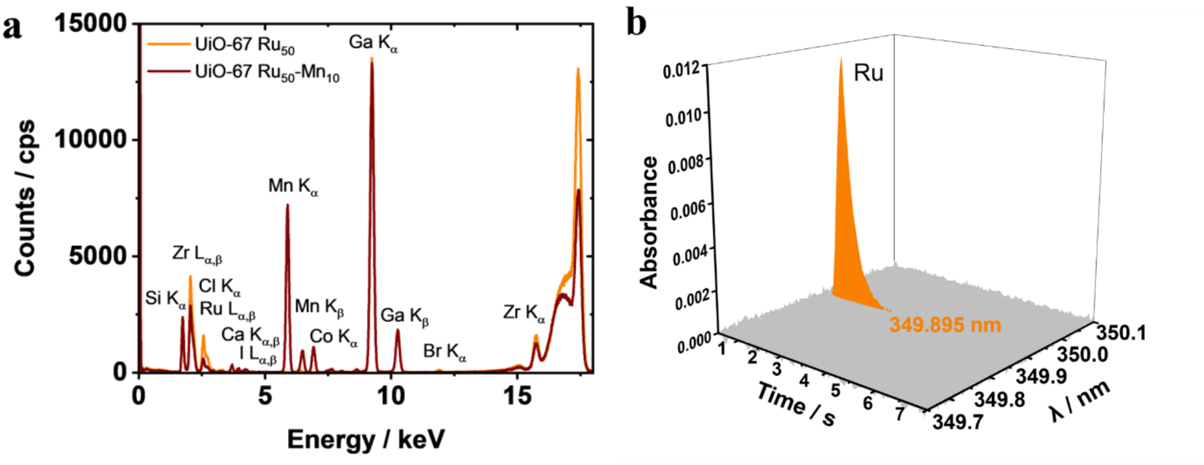

Figure S10. (a) TXRF spectra of UiO-67 Ru_{50} (orange) and UiO-67 $Ru_{50}Mn_{10}$ (red) with assigned element signals. (b) Exemplary 3D HR-CS-GFAAS spectrum of Ru.

Table S5. Ru and Mn ratio and concentration in UiO-67 Ru_{50} and UiO-67 Ru_{50}-Mn_{10} from TXRF and HR-CS-GFAAS analysis. Errors represent ±1 SD (n = 3), and for ratios, the uncertainty is derived from Gaussian error propagation of the resulting standard deviations.

MOFs	Mn by TXRF [mg g^{-1}]	Ru by GFAAS [mg g^{-1}]	Molar Ru to Mn ratio
UiO-67 Ru_{50}	-	23.2 ±1.0	-
UiO-67 Ru_{50}-Mn_{10}	4.92 ±0.01	9.2 ±0.1	1.02 ±0.01

Photocatalytic activity for the oxidation of benzylamine

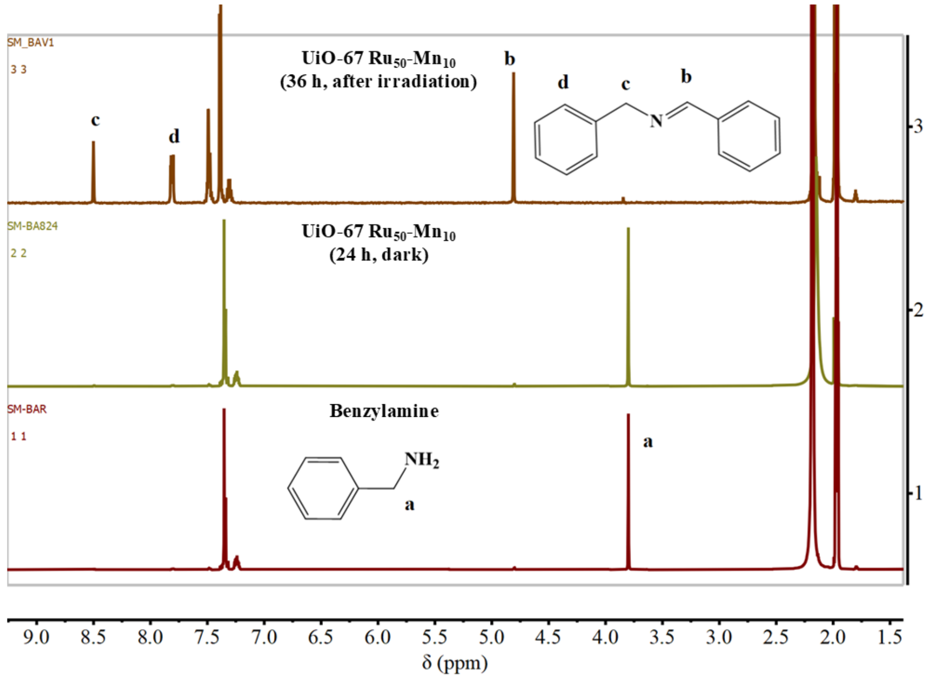

Figure S11. ^{1}H NMR spectra of the aliquots (in CD_3CN) monitored for the benzylamine oxidation reaction in the presence of UiO-67 Ru_{50}-Mn_{10} MOF under irradiation (36 h) and the dark for 24 h in a CH_3CN/H_2O mixture.

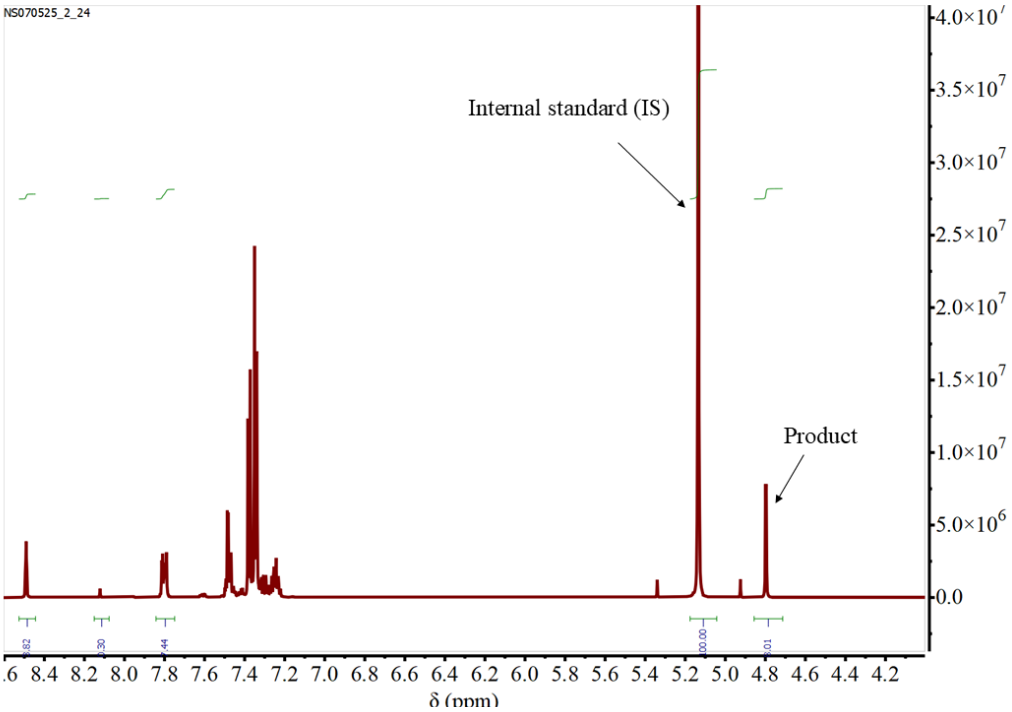

Figure S12. ^{1}H NMR spectra of the aliquots (in CD_3CN) with an internal standard collected at 24 h from the benzylamine oxidation reaction in the presence of UiO-67 Ru_{50}-Mn_{10} MOF in a CH_3CN/H_2O mixture.

Calculations of the percentage of yields and TON and TOF:

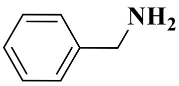

Benzylamine

Substrate

δ = **CH_2 (s, 2H, 3.80 ppm)** and ArH (m 5H, 7.51-7.14)

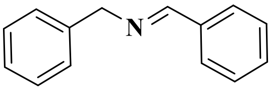

N-benzyl-1-phenylmethanimine

Product

δ = =CH (s, 1H, 8.49 ppm) **CH_2(s, 2H, 4.80 ppm)** ArH (m, 10H, 7.20-7.85 ppm)

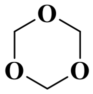

1, 3, 5 tri Oxane

Internal standard

δ = CH_2 **(s, 6H, 5.13 ppm)**

200 µL of the decant from the reaction mixture after centrifugation and evaporation at a time is mixed with 3 mg of internal standard (1,3,5-trioxane, 0.037 mmol) in CD_3CN solvent (800 µL) for ^{1}H NMR.

I_{IS} or $I_{product}$ = Integration of the proton signal for internal standards, and product at δ: 5.13 ppm, 4.80 ppm, respectively.

N_{IS} or $N_{product}$ = Number of proton signal corresponds to δ: 5.13 ppm, 4.80 ppm for internal standards and product, respectively.

The formula used for the calculation of moles of product formed:

$$\frac{n_{product}}{n_{IS}} = \frac{I_{product}}{N_{products}} \times \frac{N_{IS}}{I_{IS}}$$

No of moles of product formed in 4 mL of reaction mixture: $\frac{n_{product} \times 4\ mL}{0.2\ mL}$

No of moles of substrate (benzyl amine): 0.734 mmol in 4 mL

$$Yield\ (\%) = \frac{2 \times mol_{product}}{mol_{substrate}} \times 100$$

Table S6. Quantification of [Ru] and [Mn] in 1 mg of UiO-67 Ru and UiO-67 Ru-Mn MOFs

MOF<br>1 mg	Formula	Mol. Wt.<br>g/mole	Moles of<br>[Ru]× 10^{-6}	Moles of<br>[Mn] ×10^{-6}
UiO 67 Ru_{50}	$[Zr_6O_4(OH)_4(bpydc)_{4.724}\ (BA)_{1.13}]$ 0.70 $[Ru(bpy)_2(bpydc)]Cl_2$	2468.97	0.283	-
UiO 67 Ru_{10}	$[Zr_6O_4(OH)_4(bpydc)_{5.48}\ (BA)_{0.71}]$ 0.16 $[Ru(bpy)_2(bpydc)]Cl_2$	2208.86	0.072	-
UiO 67 Ru_{50}-Mn_{10}	$[Zr_6O_4(OH)_4(bpydc)_{3.944}\ (BA)_{1.13}]$ 0.70 $[Ru(bpy)_2(bpydc)]Cl_2$ 0.78 $[Mn(bpy)(bpydc)Cl_2]$	2679.628	0.261	0.291
UiO 67 Ru_{10}-Mn_{10}	$[Zr_6O_4(OH)_4(bpydc)_{5.30}\ (BA)_{0.71}]$ 0.16 $[Ru(bpy)_2(bpydc)]Cl_{2.}$ 0.18 $[Mn(bpy)(bpydc)Cl_2]$	2256.60	0.07	0.079

$$TON = \frac{Moles_{product}}{Moles_{Ru}}\ or\ \frac{Moles_{product}}{Moles_{Mn}}$$

$$TOF = \left(\frac{Moles_{product}}{Moles_{Ru}}\ or\ \frac{Moles_{product}}{Moles_{Mn}}\right) \times 1/time$$

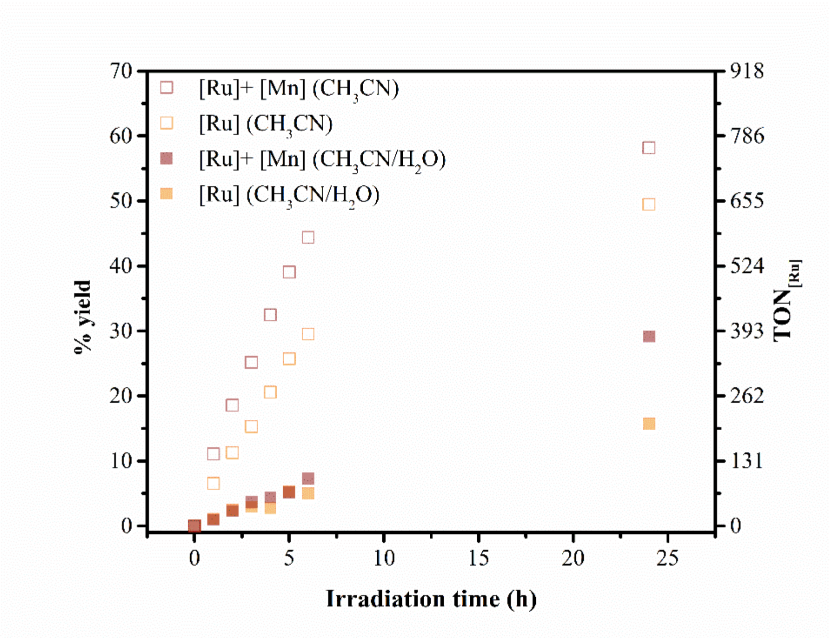

Figure S13. Activity (expressed as % yield and TON$_{[Ru]}$ *vs.* time) for the photocatalytic oxidation of benzylamine in the presence of [Ru]: [Ru(bpy)$_2$(bpydc)]Cl$_2$ alone (transparent orange) and [Ru] + [Mn] ([Ru(bpy)$_2$(bpydc)]Cl$_2$ with [Mn(bpy)$_2$Cl$_2$]·H$_2$O) (transparent wine), conducted in CH$_3$CN (hollow square) and CH$_3$CN/H$_2$O (v/v 4:1) (solid square).

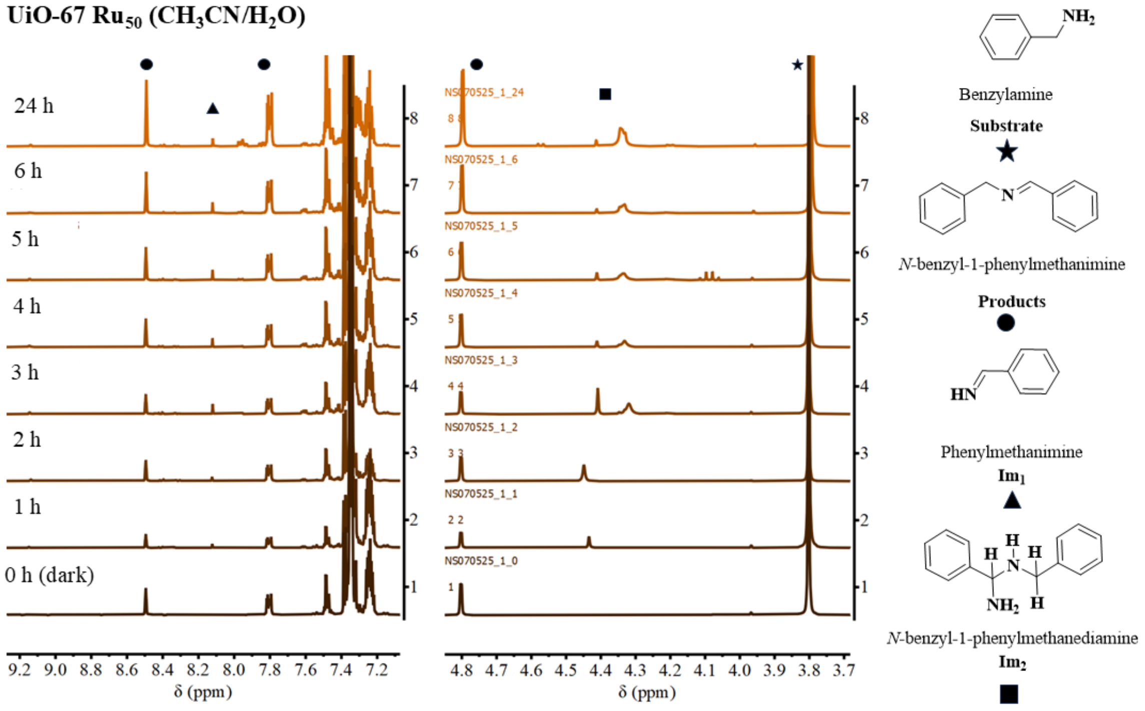

Figure S14. ^{1}H NMR spectra of the aliquots (in CD$_3$CN) collected at different time intervals during the benzylamine oxidation reaction in the presence of UiO-67 Ru$_{50}$ MOF in a CH$_3$CN/H$_2$O mixture.

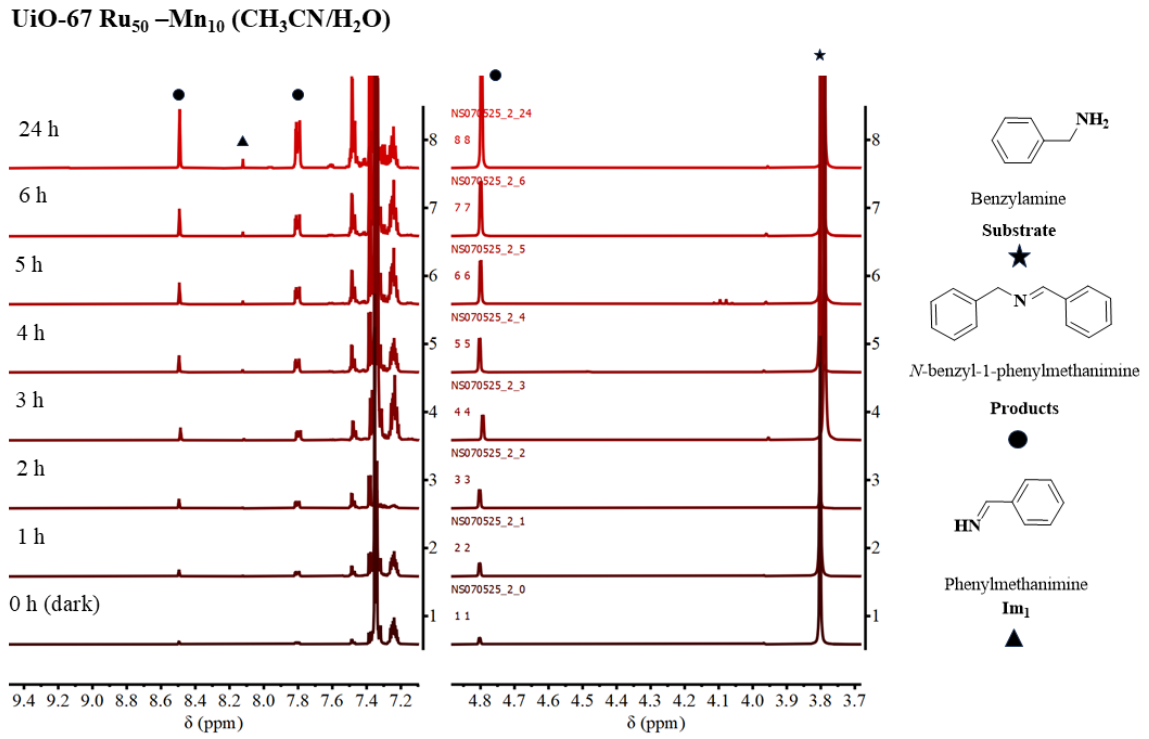

Figure S15. ^{1}H NMR spectra of the aliquots (in CD_3CN) collected at different time intervals during the benzylamine oxidation reaction in the presence of UiO-67 Ru_{50}-Mn_{10} MOF in a CH_3CN/H_2O mixture.

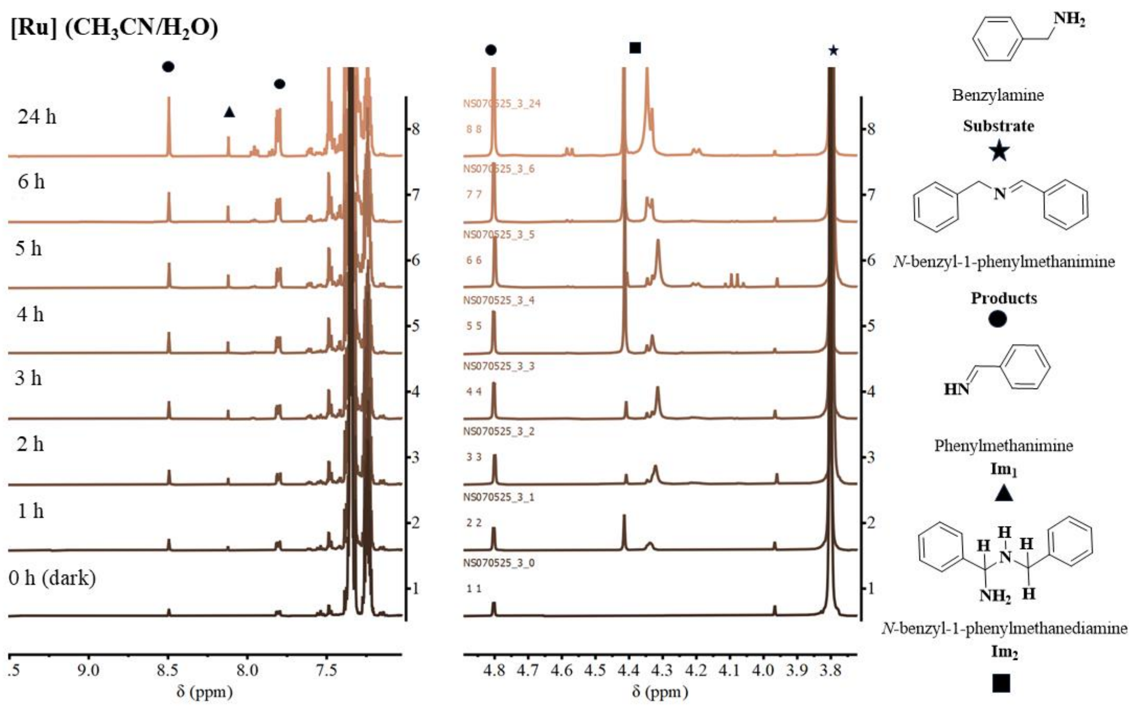

Figure S16. ^{1}H NMR spectra of the aliquots (in CD_3CN) collected at different time intervals during the benzylamine oxidation reaction in the presence of [Ru]: $[Ru(bpy)_2(bpydc)]Cl_2$ in a CH_3CN/H_2O mixture.

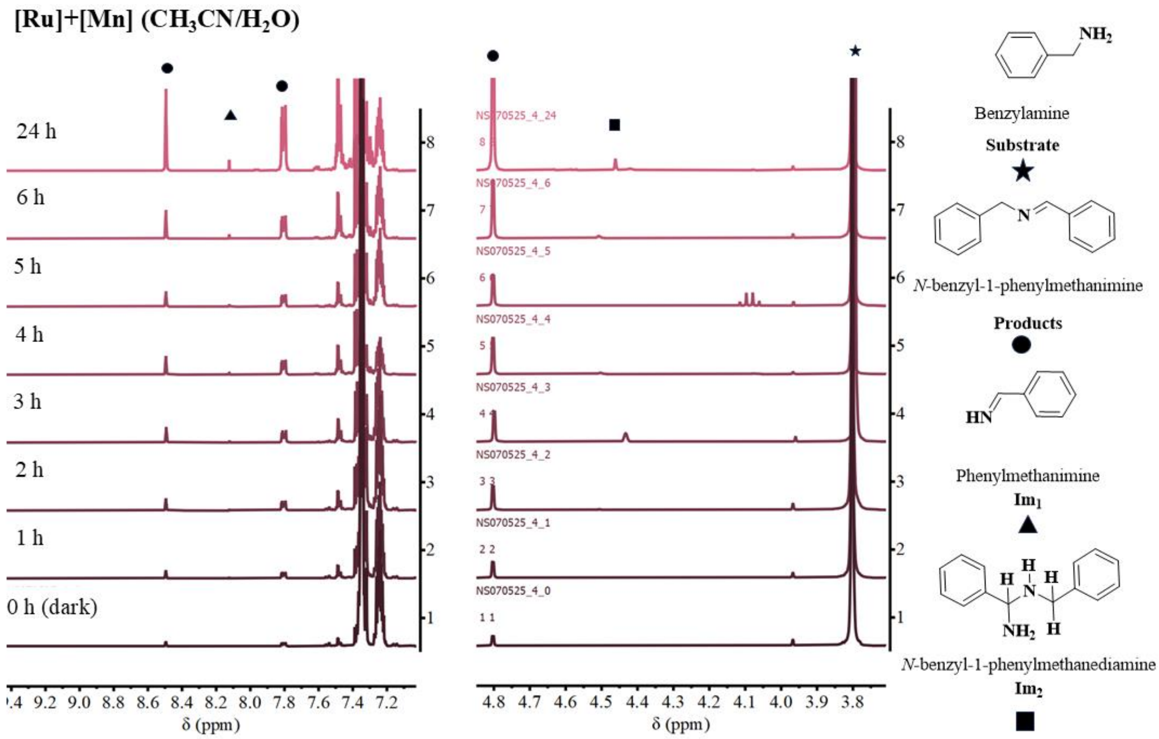

Figure S17. ^{1}H NMR spectra of the aliquots (in CD_3CN) collected at different time intervals during the benzylamine oxidation reaction in the presence of [Ru] + [Mn] ([Ru(bpy)$_2$(bpydc)]Cl_2 with [Mn(bpy)$_2$Cl$_2$]·H_2O) in a CH_3CN/H_2O mixture.

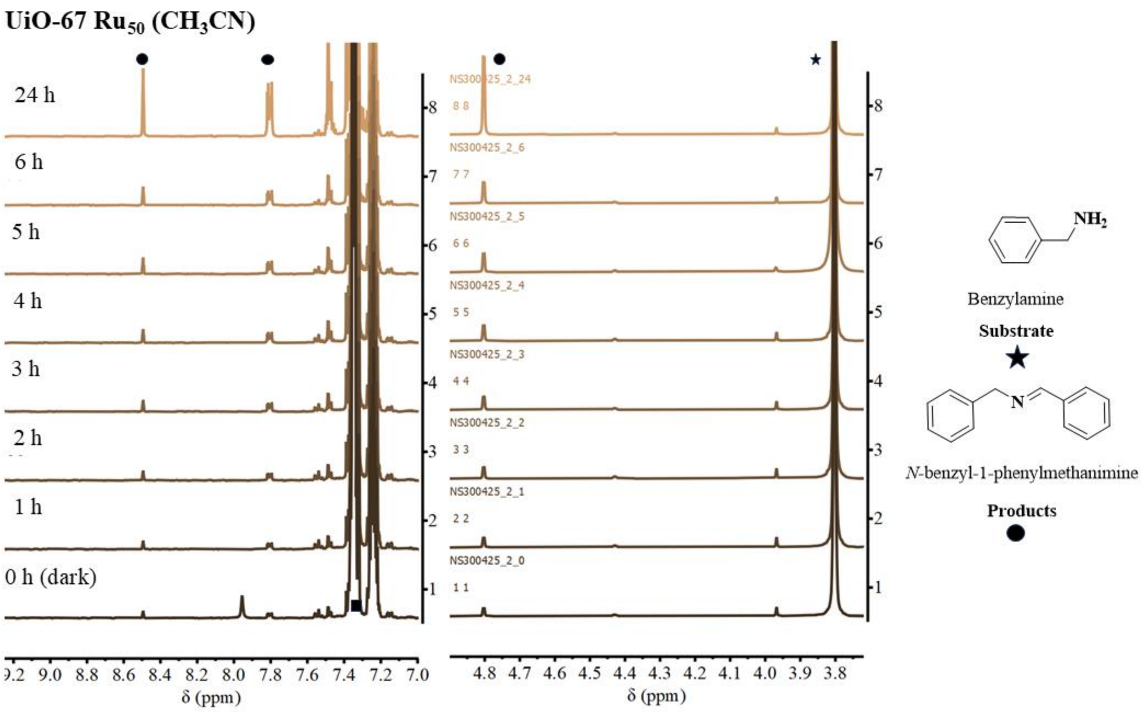

Figure S18. ^{1}H NMR spectra of the aliquots (in CD_3CN) collected at different time intervals during the benzylamine oxidation reaction in the presence of UiO-67 Ru_{50} MOF in CH_3CN.

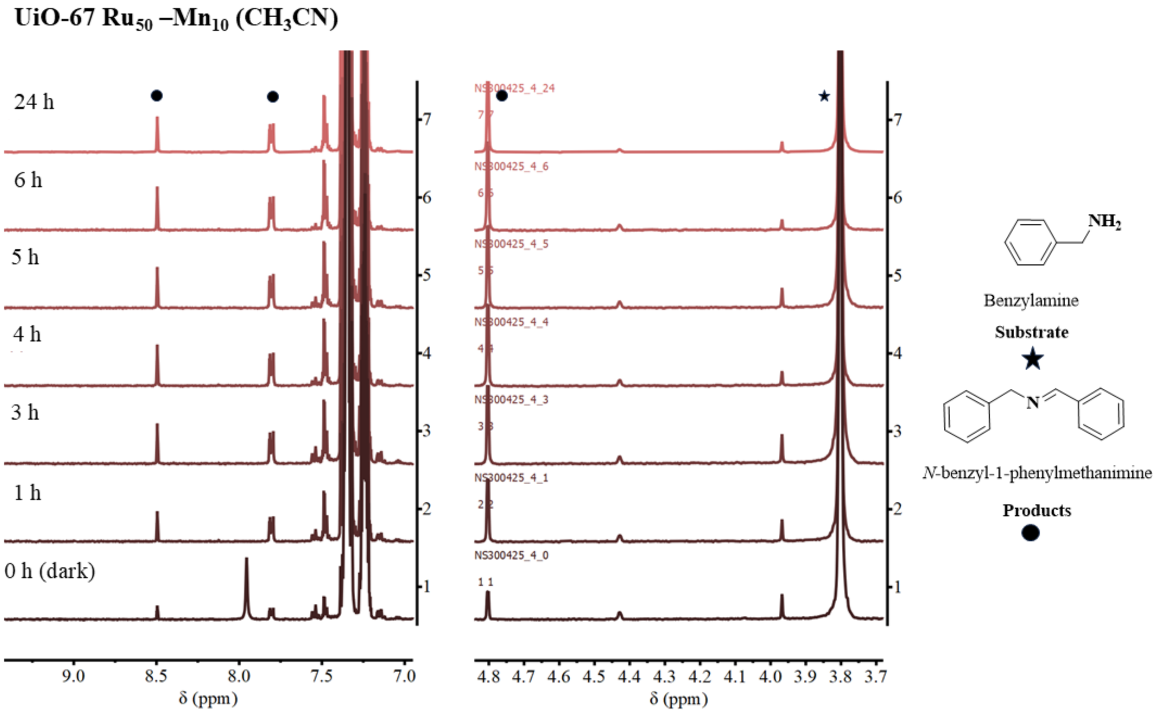

Figure S19. ^{1}H NMR spectra of the aliquots (in CD_3CN) collected at different time intervals during the benzylamine oxidation reaction in the presence of UiO-67 Ru_{50}-Mn_{10} MOF in CH_3CN.

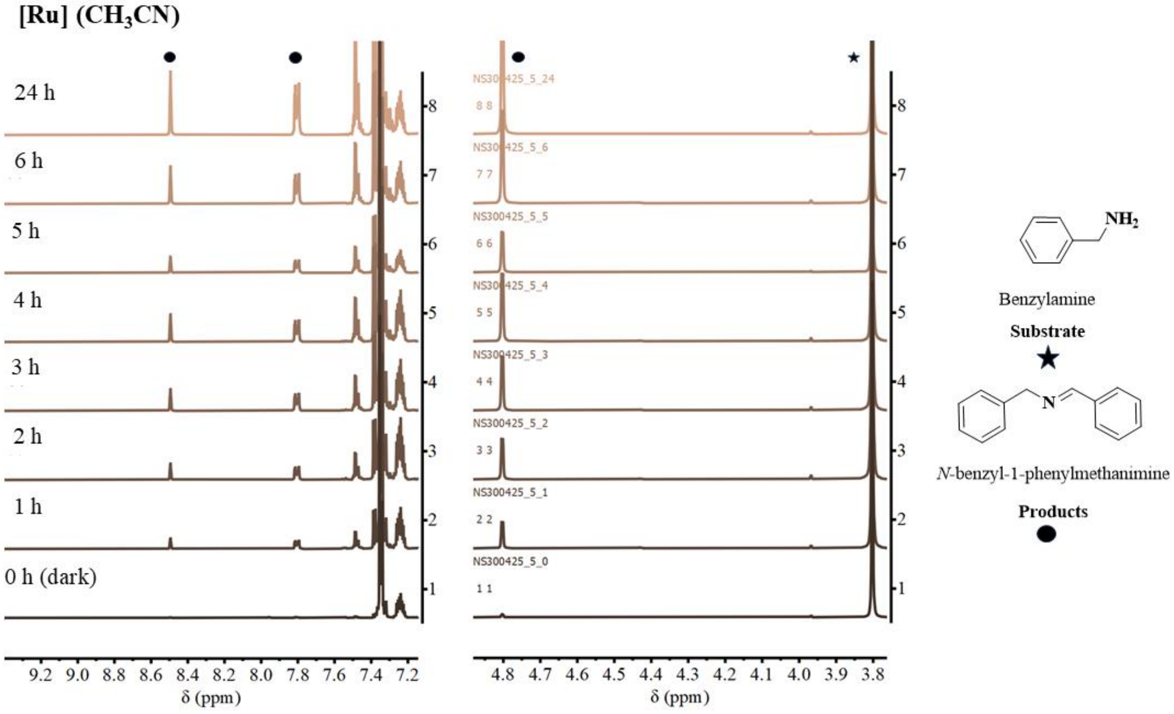

Figure S20. ^{1}H NMR spectra of the aliquots (in CD_3CN) collected at different time intervals during the benzylamine oxidation reaction in the presence of [Ru]: $[Ru(bpy)_2(bpydc)]Cl_2$ in CH_3CN.

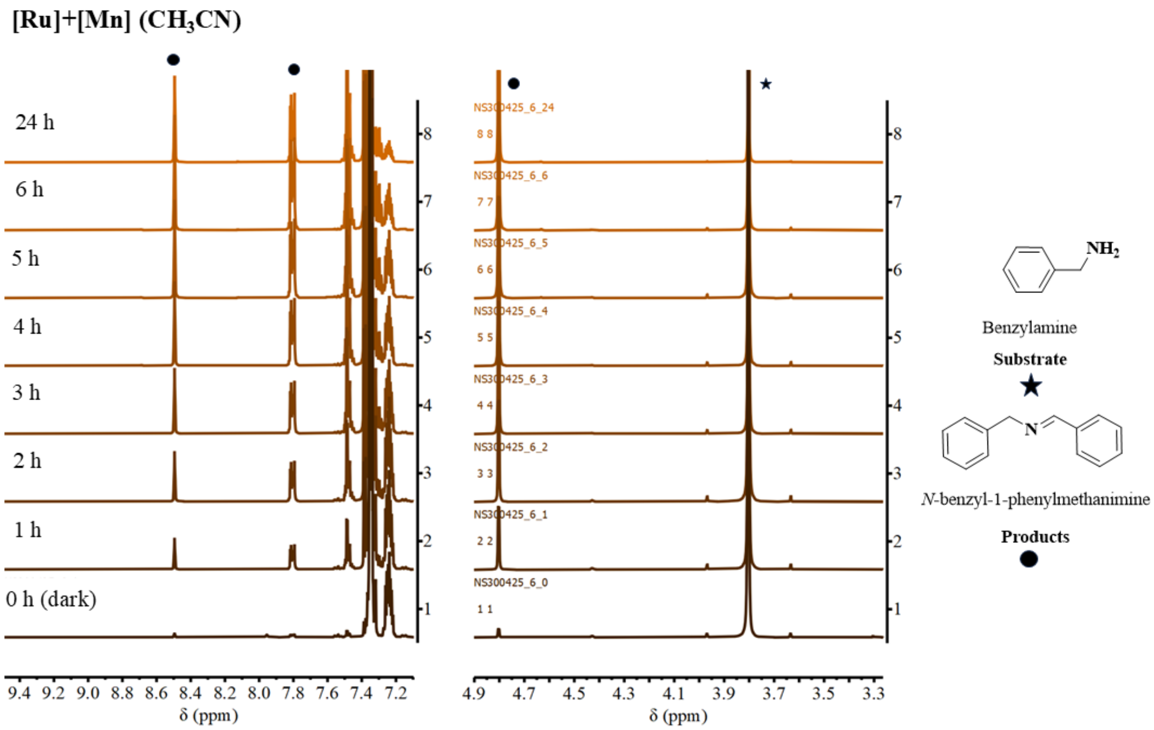

Figure S21. ¹H NMR spectra of the aliquots (in CD_3CN) collected at different time intervals during the benzylamine oxidation reaction in the presence of [Ru] + [Mn] ($[Ru(bpy)_2(bpydc)]Cl_2$ with $[Mn(bpy)_2Cl_2]\cdot H_2O$) in CH_3CN.

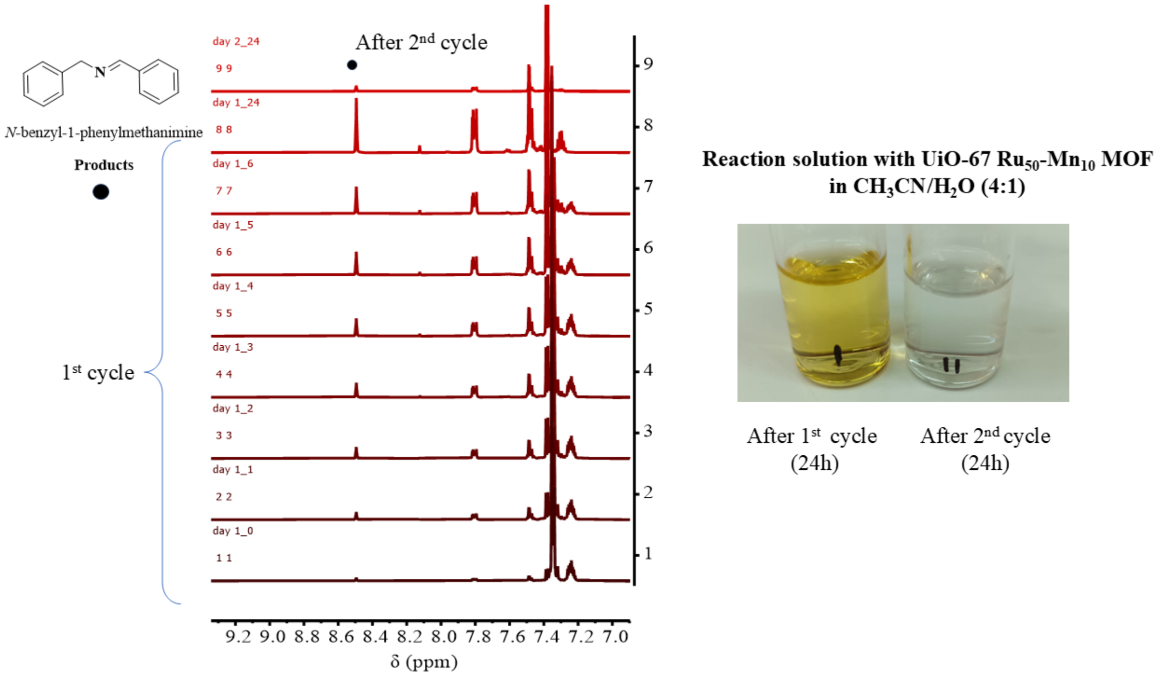

Figure S22. Recyclability test of 1 mg of UiO-67 Ru_{50}-Mn_{10} in 4 mL of CH_3CN/H_2O (4:1): (left) ¹H NMR spectra of the aliquots (in CD_3CN) collected at different time intervals (0-24h, 1st cycle) and after 2nd cycle (24 h) during the benzylamine oxidation reaction. (λ: 460 nm LED source. (Right) Pictures showing the reaction solution after the 1st and the 2nd cycle. After the 1st cycle, the solid MOF was almost dissolved (as evident by a transparent yellow solution). The residual solid recovered after the 1st cycle by

centrifugation at 7000 rpm was washed with CH_3CN (twice), and re-irradiated with the fresh 0.734 mmol of benzylamine in 4 mL of CH_3CN/H_2O (4:1) for 24 h.

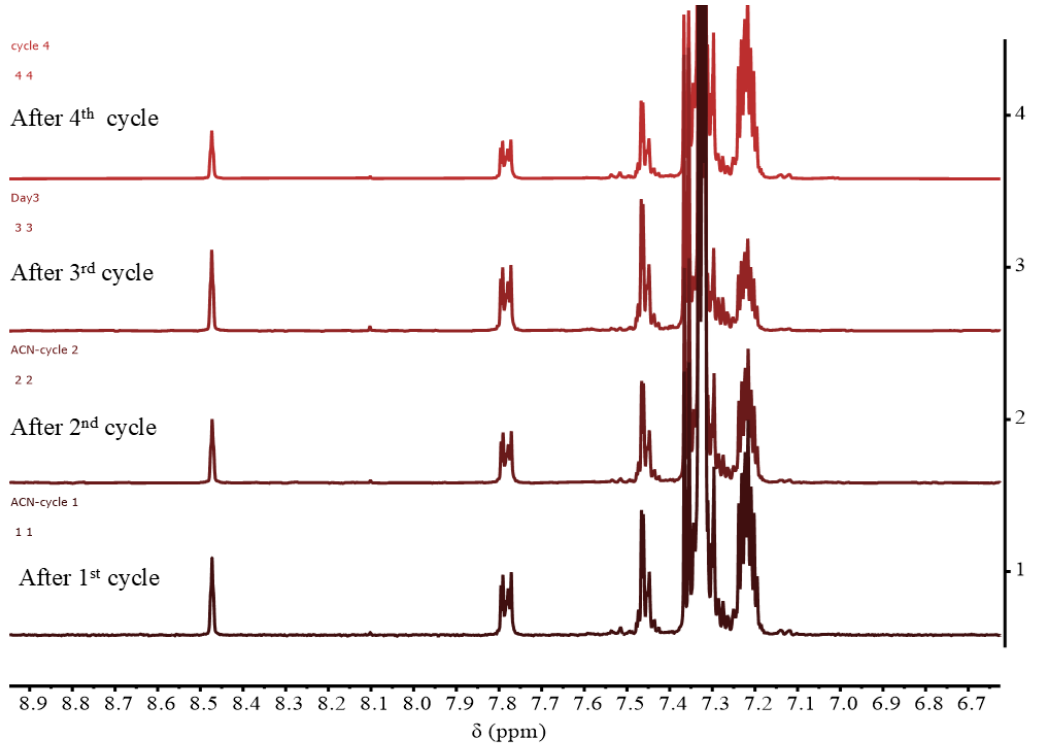

Figure S23. ^{1}H NMR spectra of the aliquots (in CD_3CN) collected at 24h (after each cycle) during the benzylamine oxidation reaction in CH_3CN (reaction condition: 1 mg of UiO-67 Ru_{50}-Mn_{10}, λ: 460 nm LED source). After each cycle (24 hours of irradiation), the solid MOF was recovered by centrifugation at 7000 rpm, washed with CH_3CN (twice), and re-irradiated with fresh 0.734 mmol of benzylamine in 4 mL of CH_3CN.

Spectroscopic studies

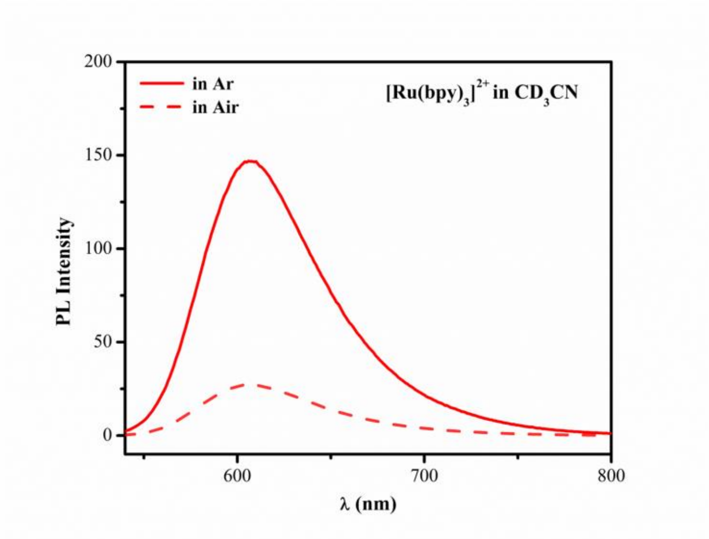

Figure S24. PL spectra of 500 µM $[Ru(bpy)_3]^{2+}$ reference solution in CD_3CN, monitored in the 550-800 nm region under both air and Ar atmospheres (λ_{ex} = 450 nm).

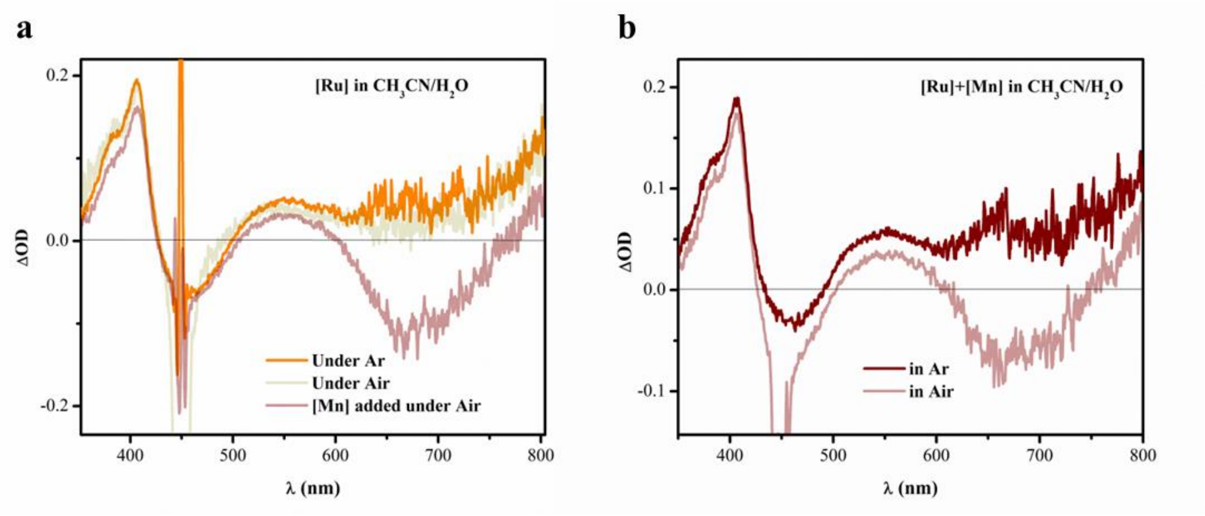

Figure S25. (a) ns-transient absorption spectra of 500 μM [Ru] complex ([Ru(bpy)$_2$(bpydc)]Cl$_2$) at a time delay of 100 ns under (1) Ar, (2) under air, and (3) in the presence of 500 μM [Mn] ([Mn(bpy)$_2$Cl$_2$]·H$_2$O) under air in CH$_3$CN/H$_2$O (4:1 v/v), acquired at λ_{pump} = 450 nm. (b) ns-transient absorption spectra of 500 μM [Ru] and 500 μM [Mn] complexes at a time delay of 100 ns (1) under Ar and (2) under air in CH$_3$CN/H$_2$O (4:1 v/v), acquired at λ_{pump} = 450 nm.

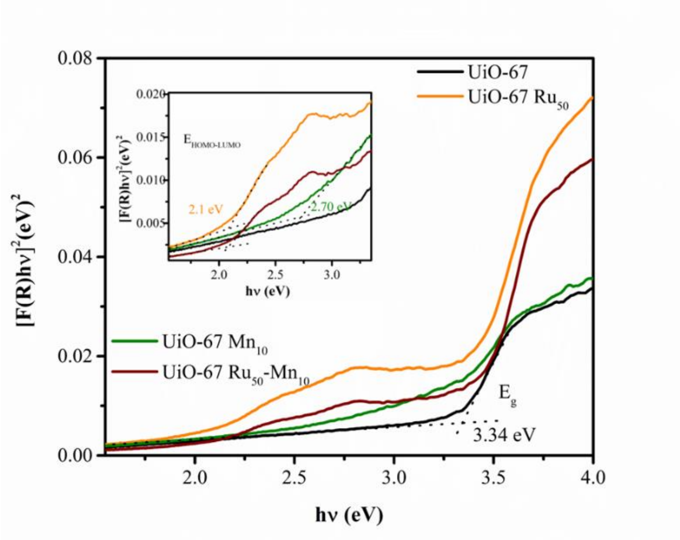

Figure S26. Tauc plots of UiO-67, UiO-67 Ru$_{50}$, UiO-67 Ru$_{50}$-Mn$_{10}$, UiO-67 Mn$_{10}$ acquired from the diffuse reflectance spectroscopy by applying the Kubelka-Munk function $F(R_\infty) = (1 - R_\infty)^2/2R_\infty$ (where $F(R_\infty)$ is reflectance of an infinitely thick specimen) of the solid MOFs.

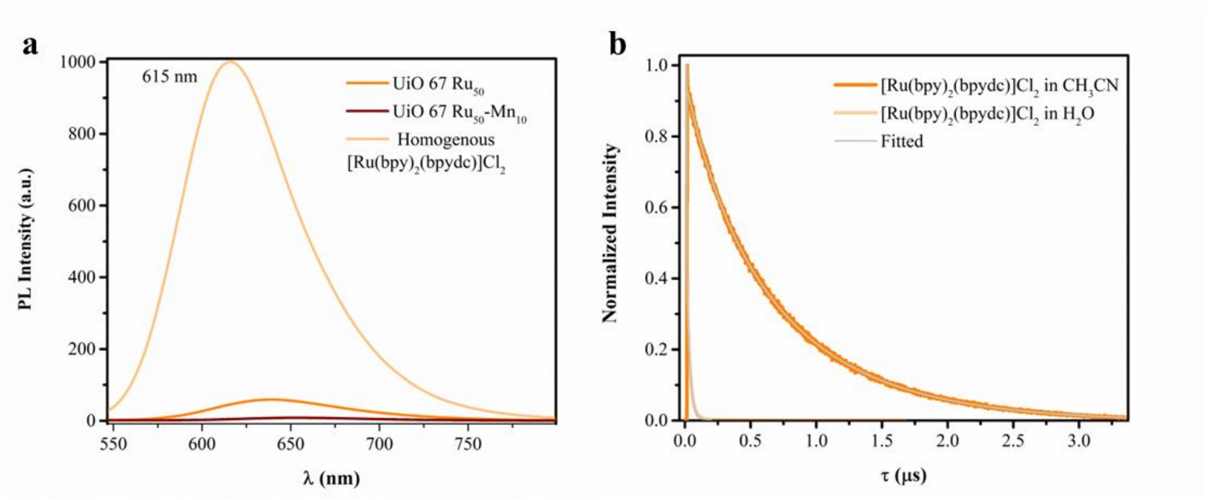

Figure S27. (a) PL (λ_{ex} = 450 nm) and (b) time-resolved PL (λ_{ex} = 372 nm with a 495 nm long pass filter) spectra of 50 μM of $[Ru(bpy)_2(bpydc)]Cl_2$ in deaerated CH_3CN and H_2O.

Table S7. The multiexponential fitting parameters (biexponential) were derived from time-resolved emission spectroscopy (triplet state decay).

Compounds	A_i			x_0	τ_i *(ns)*			Adjacent R^2	τ_{av} [b](ns)
	A_1	A_2	A_3		τ_1	τ_2	τ_3		
$[Ru(bpy)_2(bpydc)]Cl_2$	0.7265	0.1819	-	0.032	791.74±4.78	271.83±6.53	-	0.99956	750
$[Ru(bpy)_2(bpydc)]Cl_2$ [a]	0.4316	-	2.89	0.014	23.08±0.069	-	0.196±0.004	0.99931	23
UiO-67 Ru_{50}	0.2809	0.1559	659.7	0.011	262.86±0.90	39.83±0.54	0.511±0.006	0.99961	246
UiO-67 Ru_{50}-Mn_{10}	0.1684	0.3290	109.2	0.012	71.81±0.60	6.34±0.12	0.343±0.005	0.995	62
UiO-67 Ru_{50}[a]	0.1786	0.1699	2331	0.012	122.54±0.64	14.43±0.24	0.288±0.003	0.997	112
UiO-67 Ru_{50}-Mn_{10}[a]	0.1244	0.2093	33661	0.012	54.81±0.58	6.44±0.15	0.232±0.003	0.997	47

[a] measured in deaerated H_2O. The photoluminescence decay curves at 50 µM of $[Ru(bpy)_2(bpydc)]Cl_2$ alone and with 50 µM of $[Mn(bpy)_2Cl_2].H_2O$, and of UiO-67 MOFs (0.125 mg/mL) in deaerated CH_3CN. All the data were fitted to a biexponential and triexponential decay function ($y = y_0 + A_1 e^{\left(\frac{-(x-x_0)}{\tau_1}\right)} + A_2 e^{\left(\frac{-(x-x_0)}{\tau_2}\right)}$) and ($y = y_0 + A_1 e^{\left(\frac{-(x-x_0)}{\tau_1}\right)} + A_2 e^{\left(\frac{-(x-x_0)}{\tau_2}\right)} + A_3 e^{\left(\frac{-(x-x_0)}{\tau_3}\right)}$) respectively, including scattering terms for better fits. y_0= 0, and [b] τ_{av} is the average lifetime, is calculated according to the equation, $\tau_{av} = \sum_{i=1}^{2} \frac{A_i \tau_i^2}{A_i \tau_i}$. ($\tau_3$ is not considered for τ_{av} calculation as component 3 with τ_3 <expected lifetime and high amplitude attributed to scattering/background.")

Cyclic voltammetry (CV)

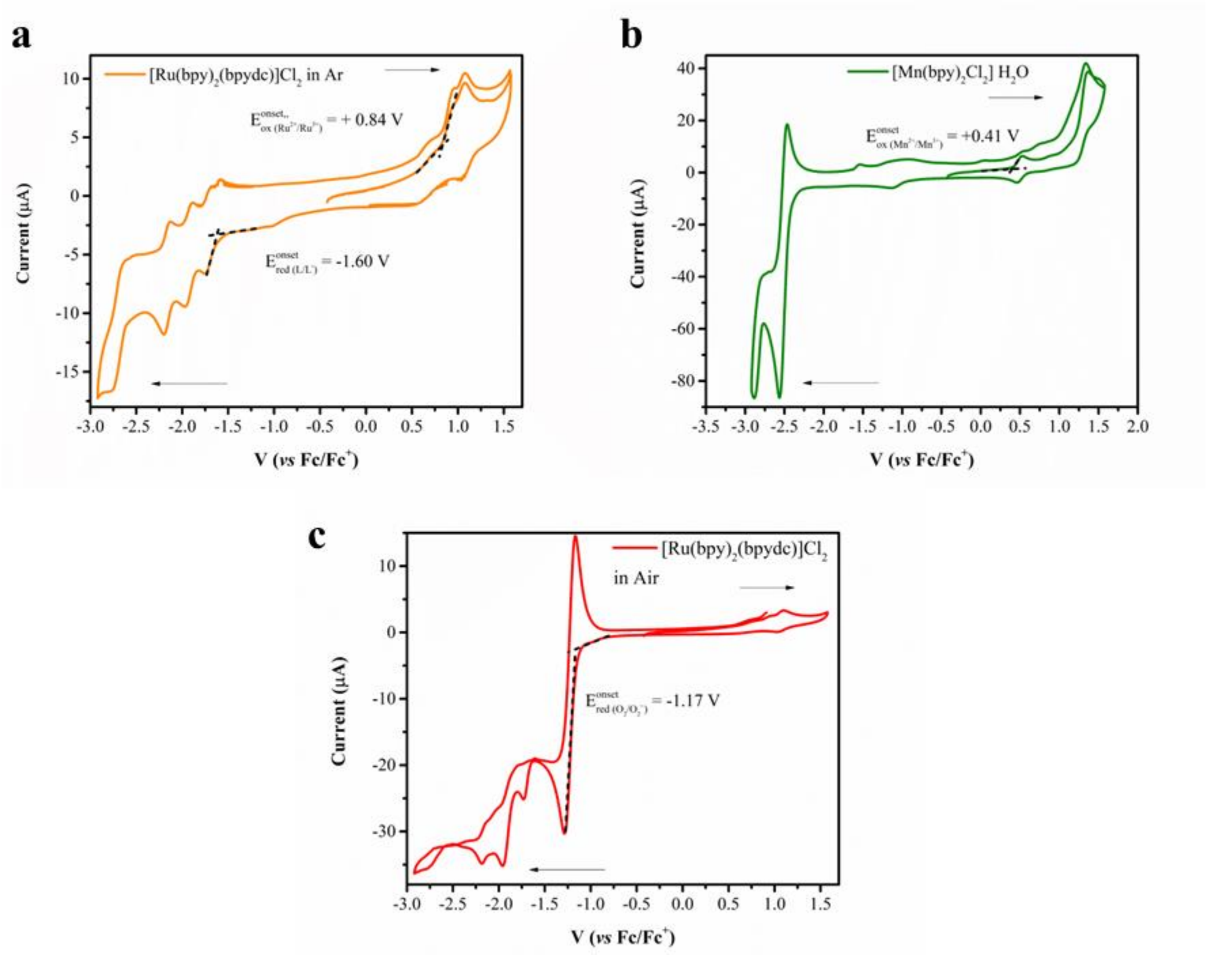

Figure S28. Cyclic voltammograms of (a) $[Ru(bpy)_2(bpydc)]Cl_2$ at 100 mVs^{-1} and (b) $[Mn(bpy)_2Cl_2].H_2O$ at 50 mVs^{-1} under Ar and (c) $[Ru(bpy)_2(bpydc)]Cl_2$ at 10 mVs^{-1} under air (Experimental conditions: CH_3CN, 0.1 M nBu_4NPF_6 supporting electrolyte, 0.25 mM analyte, glassy carbon-working electrode, Pt-wire counter electrode, Ag/AgCl quasi-reference electrode) referenced against Fc/Fc^+.

Table S8. Reductive and oxidative peaks for $[Ru(bpy)_2(bpydc)]Cl_2$ and $[Mn(bpy)_2Cl_2].H_2O$

Complex	Reductive centre	E_{red} (V *vs* NHE)	Oxidative centre	E_{ox} (V *vs* NHE)
$[Ru(bpy)_2(bpydc)]Cl_2$	Ligands	-0.91, -1.24, -1.44	Ru^{II}	+1.53
$[Mn(bpy)_2Cl_2].H_2O$	Ligand	-1.78	Mn^{II}	+1.10

The CVs of the complexes were determined in the deaerated CH_3CN solution with a scan rate of 50 mVs^{-1} and a positive initial scan direction, containing 0.1 M nBu_4NPF_6 supporting electrolyte, 0.25 mM analyte, glassy carbon-

working electrode, Pt-wire counter electrode, Ag/AgCl quasi-reference electrode. referenced against Fc/Fc^+. The potential values are given to NHE using the formula given below.

Energy level of HOMO and LUMO in $[Ru(bpy)_2(bpydc)]Cl_2$, and reduction potential of $[Mn(bpy)_2Cl_2].H_2O$ and O_2 (*vs* NHE):

According to the results of cyclic voltammetry, and the steady state absorption spectroscopy the HOMO, LUMO orbits are approximated by the following formulas:[9,10]

$$E_{Fc/Fc+}\ \textit{vs}.\ \text{NHE} = E_{Fc/Fc+}\ \text{vs. SCE} + 0.241\ \text{V} = 0.45\ \text{V} + 0.24\ \text{V} = 0.69\ \text{V}$$

Therefore,

E_{LUMO} *vs* NHE = ($E_{onset\text{-}red}$ *vs* Fc/Fc^+ + 0.69) = (-1.60 *vs* Fc/Fc^+ + 0.69) = **-0.91 V *vs* NHE**

$$E_{HOMO\text{-}LUMO}\ (\text{optical}) = 1240/\lambda_{MLCT\ (edge)} = 1240/506\ \text{eV} = 2.45\ \text{eV}$$

E_{HOMO} *vs* NHE = ($E_{HOMO\text{-}LUMO}$ (optical) + E_{LUMO} *vs* NHE) = (2.45 - 0.91) = **+ 1.54 V *vs* NHE**
= + 0.85 V (*vs* Fc/Fc^+) ~ + 0.84 V (vs Fc/Fc^+) measured in CV

Whereas, estimated potential for oxidation of the ($Mn^{II/III}$) in $[Mn(bpy)_2Cl_2].H_2O$

($E_{onset\text{-}ox}$ *vs* Fc/Fc^+ + 0.69) = (+ 0.41 *vs* Fc/Fc^+ + 0.69) = **+1.10 V *vs* NHE**.

Whereas, estimated potential for reduction of the $\mathbf{O_2/O_2^{\cdot-}}$ ($E_{onset\text{-}red}$ *vs* Fc/Fc^+ + 0.69) = (-1.17 *vs* Fc/Fc^+ + 0.69) = **-0.48 V *vs* NHE**

Whereas, literature values of benzyl amine to imine: +0.76 V *vs* NHE [11,12]

Quantification of H_2O_2 using the $TiOSO_4$ method

Hydrogen peroxide (H_2O_2) was quantified using the $TiOSO_4$ method, as previously reported in the literature.[13–15] A $TiOSO_4$ stock solution was prepared by dissolving 0.1 M $TiOSO_4$ in 2 M H_2SO_4. For the photocatalytic reaction, 1 mg of UiO-67 Ru_{50}–Mn_{10} was dispersed in 4 mL of CH_3CN and irradiated under aerobic conditions for 24 h using an LED source in an Azula photoreactor. After irradiation, 100 µL of the reaction mixture was withdrawn and centrifuged. The clear supernatant was then transferred into a quartz cuvette, mixed with 500 µL of $TiOSO_4$ solution, and subsequently diluted with 2.5 mL of deionized water. Upon addition of the reaction mixture to the $TiOSO_4$ solution, a visible color change from colorless to yellow was observed, which is attributed to the formation of pertitanic acid.

The absorbance of the resulting solution was recorded at 407 nm using a UV–Vis spectrophotometer. The molar extinction coefficient (ε) at 407 nm was taken as 6.89×10^2 M^{-1}

cm^{-1}.[16] Control experiments were conducted to validate the measurements: the UV–Vis spectra of (i) the photocatalytic reaction solution without $TiOSO_4$ and (ii) freshly prepared $TiOSO_4$ solution were measured under identical conditions. In both cases, no significant absorption in the 400–450 nm region was detected. Based on the calibration and absorbance data, the concentration of H_2O_2 formed in the reaction system was determined to be 1.41×10^{-5} M.

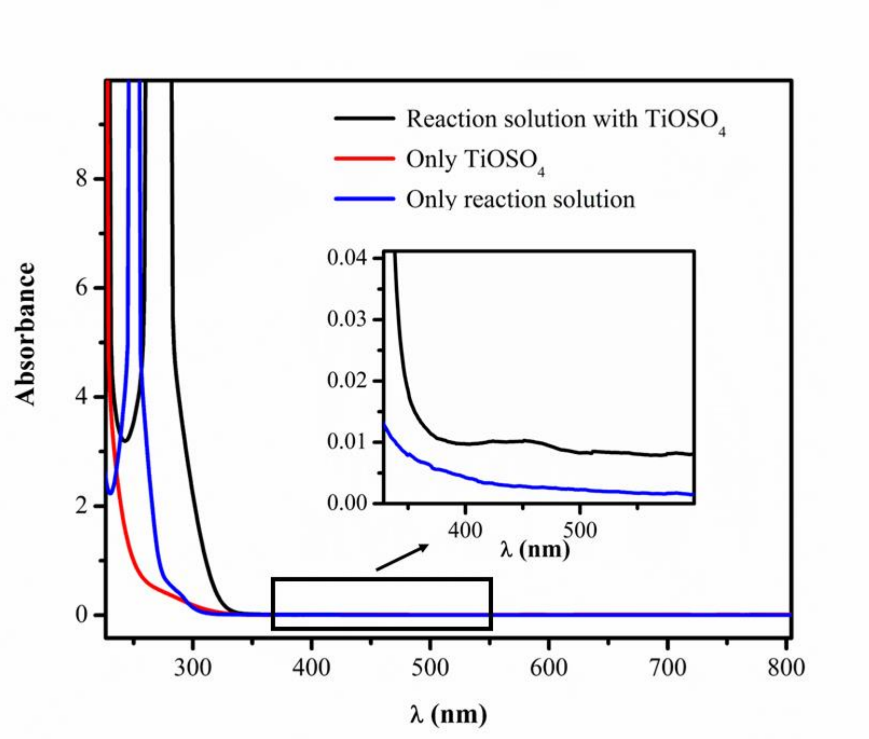

Figure S29. The UV–vis absorption spectra of (i) the photocatalytic reaction solution with $TiOSO_4$, (ii) freshly prepared $TiOSO_4$ solution, and (iii) only the photocatalytic reaction solution. The enlarged spectra at the selected wavelength region are shown in the inset.

References

1 R. Navarro Amador, M. Carboni and D. Meyer, Sorption and photodegradation under visible light irradiation of an organic pollutant by a heterogeneous UiO-67–Ru–Ti MOF obtained by post-synthetic exchange, *RSC Adv*, 2017, **7**, 195–200.

2 N. Saravanan, P. Mayuri, S.-T. Huang and A. S. Kumar, electrochemical immobilization of [Mn(bpy)2(H2O)2]2+ complex on MWCNT modified electrode and its electrocatalytic H2O2 oxidation and reduction reactions: A Mn-Pseudocatalase enzyme bio-mimicking electron-transfer functional model, *Journal of Electroanalytical Chemistry*, 2018, **812**, 10–21.

3 S. Rothbart, E. Ember and R. van Eldik, Comparative study of the catalytic activity of [MnII(bpy)2Cl2] and [Mn2III/IV(μ-O)2(bpy)4](ClO4)3 in the H2O2 induced oxidation of organic dyes in carbonate buffered aqueous solution, *Dalton Transactions*, 2010, **39**, 3264.

4 Z. Li, D. Xu, J. Nie, Z. Wu, J. Wu and M. Chiang, Synthesis and Crystal Structure of Bis(2,2′-Bipyridine-N,N′)Dichloromanganese(II) Complex with Free 2,2′-Bipyridine, *J Coord Chem*, 2002, **55**, 1155–1160.

5 S. Mandal, R. Leiter, J. Biskupek, U. Kaiser and A. Pannwitz, The Zr_8O_6 Secondary Building Unit and Porphyrin Linker Catalyze Light-Driven H_2 Evolution in Porphyrin-Based Metal Organic Frameworks, *ChemSusChem*, DOI:10.1002/cssc.202500372.

6 W. A. Maza and A. J. Morris, Photophysical Characterization of a Ruthenium(II) Tris(2,2′-bipyridine)-Doped Zirconium UiO-67 Metal–Organic Framework, *The Journal of Physical Chemistry C*, 2014, **118**, 8803–8817.

7 C.-C. Hou, T.-T. Li, S. Cao, Y. Chen and W.-F. Fu, Incorporation of a $[Ru(dcbpy)(bpy)_2]^{2+}$ photosensitizer and a $Pt(dcbpy)Cl_2$ catalyst into metal–organic frameworks for photocatalytic hydrogen evolution from aqueous solution, *J Mater Chem A Mater*, 2015, **3**, 10386–10394.

8 J. Zhou, S. Gu, Y. Xiang, Y. Xiong and G. Liu, UiO-67: A versatile metal-organic framework for diverse applications, *Coord Chem Rev*, 2025, **526**, 216354.

9 D. A. Jose, A. D. Shukla, G. Ramakrishna, D. K. Palit, H. N. Ghosh and A. Das, Physicochemical and Photophysical Studies on Porphyrin-Based Donor−Acceptor Systems: Effect of Redox Potentials on Ultrafast Electron-Transfer Dynamics, *J Phys Chem B*, 2007, **111**, 9078–9087.

10 H.-C. Chen, J. N. H. Reek, R. M. Williams and A. M. Brouwer, Halogenated earth abundant metalloporphyrins as photostable sensitizers for visible-light-driven water oxidation in a neutral phosphate buffer solution, *Physical Chemistry Chemical Physics*, 2016, **18**, 15191–15198.

11 H. Liu, C. Xu, D. Li and H. Jiang, Photocatalytic Hydrogen Production Coupled with Selective Benzylamine Oxidation over MOF Composites, *Angewandte Chemie International Edition*, 2018, **57**, 5379–5383.

12 X. Yang, T. Huang, S. Gao and R. Cao, Boosting photocatalytic oxidative coupling of amines by a Ru-complex-sensitized metal-organic framework, *J Catal*, 2019, **378**, 248–255.

13 K. Wenderich, B. A. M. Nieuweweme, G. Mul and B. T. Mei, Selective Electrochemical Oxidation of H_2O to H_2O_2 Using Boron-Doped Diamond: An Experimental and Techno-Economic Evaluation, *ACS Sustain Chem Eng*, 2021, **9**, 7803–7812.

14 B. J. Deadman, K. Hellgardt and K. K. (Mimi) Hii, A colorimetric method for rapid and selective quantification of peroxodisulfate, peroxomonosulfate and hydrogen peroxide, *React Chem Eng*, 2017, **2**, 462–466.

15 G. Eisenberg, Colorimetric Determination of Hydrogen Peroxide, *Industrial & Engineering Chemistry Analytical Edition*, 1943, **15**, 327–328.

16 Z. Machala, B. Tarabova, K. Hensel, E. Spetlikova, L. Sikurova and P. Lukes, Formation of ROS and RNS in Water Electro-Sprayed through Transient Spark Discharge in Air and their Bactericidal Effects, *Plasma Processes and Polymers*, 2013, **10**, 649–659.
